# Supplementary material for: Zygotic activation of transposable elements during zebrafish early embryogenesis
Source: Nat Commun. 2025 Apr 18;16:3692. doi: 10.1038/s41467-025-58863-7 (PMC12006353; doi:10.1038/s41467-025-58863-7)
Supplement: Supplementary file 1 — Supplementary information [file 41467_2025_58863_MOESM1_ESM.pdf]

Supplementary Note

1. Manual curation on TE-alone transcripts and loci

Due to the repetitive nature of transposable elements, the CD-HIT strategy is not well-suitable for merging highly identical TE-alone transcripts, i.e., transcripts from different loci can be inappropriately merged. Therefore, we adopt manual annotation to refine TE-alone transcripts to achieve the highest accuracy on TE annotation. We perform stringent manual curation on TE-alone transcripts to reduce inaccurate annotation, focusing primarily on the following four areas: (i) Merge highly identical transcripts generated from the same TE-alone loci. TALON is highly sensitive in characterizing novel transcripts. By checking the long reads, corresponding transcripts, and TE-alone loci, highly identical transcripts can be manually merged into a single transcript. GT-AG splicing signal is used to determine the authenticity of an intron splicing. Sometimes, expression levels are considered, and lowly expressed transcripts are always merged into highly expressed transcripts (Supplementary Fig. 1a). (ii) Modify unreliably annotated TE-alone loci. TALON sometimes annotates a complete TE-alone locus into several smaller loci. Based on the TE annotation, these TE-alone loci can be integrated into a single locus (Supplementary Fig. 1b). Moreover, TE-alone loci structures are redefined based on the complete TE-alone transcripts and the genomic coordinates for TE-alone loci are extended based on RepeatMasker annotation, which can give out precise annotation for both TE-alone loci and TE-alone transcripts. Taking LTRs as examples, RepeatMasker annotated LTR and Internal region separately, our manual annotation can precisely define a complete LTR locus including 5'LTR, Internal sequences and 3'LTR. (iii) Adjust the structure of TE-alone transcripts. Small indels (insertion or deletions) can cause novel

transcripts to be identified. After examining the sequences of most long reads and GT-AG signal, several transcripts can be merged with other transcripts (Supplementary Fig. 1c). (iv) Remove wrongly annotated SINEs. Our pipeline outputs 16 expressed SINEs, whereas 14 SINE3-1 are also annotated as 5S rDNA loci in Ensembl (Supplementary Fig. 1d). We carefully examine these loci and find the long reads aligned here are approximately 119 bp, which are similar to the length of the zebrafish 5S rDNA genes but much shorter than the typical length of SINE3-1 (590bp for an intact copy). Due to the highly identical sequences between SINE and 5S rDNA[1], we believe these aligned reads are 5S rRNA sequences and these rDNA loci are falsely annotated as SINEs and therefore remove them from our TE annotation. Only two SINE transcripts are real but with low expression. Ultimately, these steps generate a high-confidence TE annotation for 706 TE-alone transcripts and 550 active TE-alone loci.

2. Characterization of chimeric TE-alone transcripts

Although a majority of TE-alone transcripts are generated from single TE-alone loci, 48 chimeric TE-alone transcripts (transcripts generated from more than two nearby TE-alone loci, which are classified into different TE subfamilies) are also found and characterized into major and minor TE components based on sequence proportion (Supplementary Fig. 3a). This analysis can help us in how to classify these chimeric transcripts, where we refer to the TE type/family/subfamily of major components for the assignment. Most (38/48) chimeric TE-alone transcripts are comprised of only two different kinds of TEs, while one has a very complicated structure (Supplementary Fig. 3b,c). For the formation of chimeric TE-alone_transcripts, we find 81.25% are more likely formed by improper transcription

termination or readthrough transcription[2]. The remaining nine chimeric transcripts are generated by nested TE insertion (Supplementary Table 1 and Supplementary Fig. 3c).

3. Comparison between short-read and long-read RNA-seq

We establish two pipelines to directly evaluate the performance of short-read and long-read RNA-seq data in identifying TE-alone transcripts (Supplementary Fig. 4a). We identified 9,741 and 1,817 TE-alone transcripts with short-read and long-read RNA-seq data for zebrafish, and 25,935 and 1,448 for mouse (Supplementary Fig. 4b). Short-read RNA-seq results are consistent with previous studies, which typically find thousands to tens of thousands of expressed TE-alone copies[3,4], much higher than those from long-read data. The overall comparison of transcript length indicates significantly shorter length for transcripts defined by short-read RNA-seq data (Supplementary Fig. 4c). Moreover, short-read RNA-seq data often assembles multiple short transcripts, which are further confirmed as fragmented transcripts from single complete transcripts by long-read data (Supplementary Fig. 4d). Mapping errors occur quite often for short-read alignment (Supplementary Fig. 4d). Importantly, the TE-alone loci identified by short-read RNA-seq data are not largely overlapped with those from long-read RNA-seq data, suggesting that a large amount of expressed TE-alone loci identified by short-read data may not be the true expressed copies since the aligned locations are randomly selected from multiple best alignments (Supplementary Fig. 4e). Therefore, we conclude that long-read RNA-seq technology can both dramatically reduce the number of expressed TE-alone loci and find the truly expressed TE-alone copies, compared with short-read data. In addition, long-read RNA-seq can provide full-length TE-alone transcripts, which can greatly facilitate further TE-alone structure annotation.

4. Intron origination by TE insertion

TE insertion has been proposed as an avenue for intron origination[5]. We observe multiple lines of evidence to support this theory. Most LINEs do not contain introns according to our analysis. However, a few multi-exon LINE transcripts clearly demonstrate that these introns are acquired from the insertion of other kinds of TEs (Supplementary Fig. 5c). Similar evidence is also found from intron-containing LTR transcripts (Supplementary Fig. 5c), indicating that TE insertion can be involved with intron evolution. To know whether these external TE fragments are removed in a manner of intron splicing, we inspect the 189, 155 and 12 splicing sites, and find 187, 112 and 5 obey the “GU..AG” splicing rule for DNA transposons, LTRs and LINEs, respectively (Supplementary Fig. 5a), implying these inserted TE regions are removed as intron splicing mechanism[6].

5. Identifying active TE-alone transcripts during zygotic genome activation

Maternal TE-alone transcripts are defined if TPM > 0 in either fertilized egg or the 1-cell stage, otherwise the transcripts are defined as newly activated transcripts. Subsequently, 11 maternally deposited and 695 newly expressed TE-alone transcripts are characterized. We identify four putative newly activated TE-alone transcripts at the 64-cell stage (Supplementary Fig. 6b). However, all have only one supportive read and three of them are DNA transposons. Moreover, three transcripts are generated from known loci annotated as genes in Ensembl. Therefore, we do not recognize the 64-cell stage as the initial stage for TE activation due to the extremely low expression and potential domesticated TEs. Based on the expression pattern of TE-alone transcripts, we define the zygotic TE activation (ZTA) begins at the 1k-cell stage and becomes prevalent at the oblong stage, supported by both long-read RNA-seq and WISH experiment (Fig. 2a,c and

Supplementary Fig. 6d). Of note, the expression of ZTA between the 64-cell and 1k-cell stages and between the high and oblong stages showed the lowest correlation. Similarly, the expression of ZGA between the 1-cell and 64-cell and between the 64-cell and 1k-cell stages (corresponding to the minor and major waves of ZGA) also showed the lowest correlation (Fig. 2b).

6. Family/subfamily-level analysis on ZTA

Family/subfamily-level expression abundances are calculated by the sum of TE-alone transcripts according to the TE family and subfamily classification and are used to describe the overall expression landscape. For seven LTR families, the overall expression patterns for most families are quite similar during ZTA, showing continuous increases from the 1k-cell to shield stages, except for two lowly expressed families (i.e., Copia and Ngaro) (Supplementary Fig. 10a). Five LINE families also show an increase of expression during ZTA; however, most are saturated around the sphere, dome or 30%-epiboly stages, earlier than LTRs to reach to the expression peaks (Supplementary Fig. 10a). Compared to LTRs and LINEs, DNA transposon families show a more complicated pattern, including some with continuously increased expression until the shield stage (e.g., CMC-EnSpm and TcMar), some with an increase from the 1k-cell or high stage to the sphere or dome stage, and a decrease until the shield stage (e.g., Kolobok-T2 and PIF-Harbinger). This extensive variation is more obvious for the lowly expressed families (Supplementary Fig. 10a).

This expression heterogeneity is extended to the subfamily-level observation, not only for the subfamilies from different families, but also for subfamilies from the same families (Supplementary Fig. 10b). For example, 20 DNA transposon subfamilies of CMC-EnSpm

family are mainly clustered into four subgroups, showing different activation times, peak stages, and expression patterns (Supplementary Fig. 10b). These results highlight previously reported distinct expression patterns among TE families/subfamilies[8,9].

7. Comparative genomic analysis on active TE-alone loci

To determine the evolutionary ages for these active TE-alone loci, we conduct a comparative genomic analysis on TU reference genome with three other genome assemblies from different zebrafish strains: AB, Nadia, and CB (Supplementary Fig. 14a). By extracting 25 kb upstream and downstream regions of individual TE-alone locus as query sequences, we identify orthologous regions from these three genomes (Supplementary Fig. 14a). This analysis leads us to discover 335 TE-alone loci only existing in TU, 172 shared by TU and AB, 13 shared by TU, AB and Nadia and 14 shared by all four genomes (Supplementary Fig. 14b), based on a previously established phylogeny[10]. In addition, 16 TE-alone loci are classified into other types, i.e., shared by TU and Nadia or shared by TU and CB, etc. For these four groups along with the evolutionary pedigree, we can observe that evolutionarily young groups tend to be highly expressed at later stages, while older groups show higher expression (as the median) at earlier stages (Supplementary Fig. 14c,d). According to the evolutionary distance[11], we further combine TU and TU-AB groups into the “young” group, and TU-AB-Nadia and TU-AB-Nadia-CB into the “old” group, which show distinct expressions (Fig. 3h). We observe that young and old groups show distinct activation and expression patterns by testing the distribution among eight developmental stages and eight expression clusters (Supplementary Fig. 14e,f).

8. Epigenetic regulation on gene loci versus TE-alone loci

We compare multiple epigenetic landscapes between TE-alone and gene loci. For ATAC-seq data, TSSs around gene loci show typical sharp peaks, suggesting the open chromatin status (Supplementary Fig. 16a). Moreover, the overall chromatin openness continuously increases from the 256-cell to 80%-epiboly stages, consistent with the zygotic gene activation during this period[12]. The reason for different shapes of ATAC-seq between gene and TE-alone is likely due to the high frequency of multiple mapping of short reads on TE-alone regions (including TSSs), since for both LTR and LINE, the transcription start sites are mostly situated within repetitive regions, i.e., 5'LTRs.

The different tendencies of open chromatin accessibility may reflect the different regulation and activities between genes and TE-alone loci. During early embryonic development, most genes are activated and continuously expressed, while TE-alone loci have only a small window to be active and become repressive after the shield or nearby stages (Fig. 5a). Therefore, it is reasonable to see a drop in openness around the shield stage for TE-alone loci since epigenetic changes are prior to expression alteration [12]. This observation is also consistent with the profile of the histone modification, H3K9me3, which is the major repressive marker for TE silencing[13]. This marker is detected to be deposited with an elevated abundance as early as the sphere stage although the overall level is relatively low (Fig. 5d). However, there is no increased level of H3K9me3 detected on gene loci during the same stages (Supplementary Fig. 16g), indicating different regulation on genes and TEs by H3K9me3.

9. Small-RNA data analysis and piRNA-dependent silencing for TE-alone transcripts

The profiles of small-RNA data between TE-alone and gene loci are completely different (Fig. 5h and Supplementary Fig. 16h). TE-related small-RNA levels experience a rapid decrease from the 1-cell to 48 hpf stages, suggesting most are inherited maternally; while gene-related small-RNA levels undergo a dramatic increase during the transition from the 512-cell to oblong stages, in line with the miRNA activation (i.e., MiR-430) during ZGA[14]. Notably, we observe a significant increase of small-RNA levels on TE-alone transcripts from the oblong to the 50%-epiboly stage (Supplementary Fig. 16e), in line with the zygotic TE activation. In contrast, we cannot observe a similar increase in either the 1-cell to 512-cell stages (where quite a few new zygotic TE-alone transcripts are expressed) or the 6-somite to 48 hpf (where TE activation is strongly repressed). This finding implies that these newly increased small RNAs could be generated from the degradation process of the zygotically activated TE-alone transcripts by PIWI-piRNA complex, although we cannot exclude the possibility that these are the nascent piRNAs derived from piRNA loci with similar sequence context. The gene expression of *piwil1/2* and other pathway-related genes are detectable during early development (before 75%-epiboly) (Supplementary Fig. 16f), also implying this silencing pathway is effective during ZTA. Of note, the piRNA-mediated post-transcriptional regulation on TE transcription generally occurs in the cytoplasm, implying a possible connection with the subcellular localization bias of TE-derived transcripts.

We further investigate whether these TE-related small RNA populations are piRNAs or not, although previous studies suggested these small RNAs mapped on repetitive regions are piRNAs[15,16]. We first analyze the length distribution of small RNA reads that are mapped onto active TE-alone loci and find that most of the reads (ranging from 23 to 33

nt) are consistent with the typical lengths of piRNAs (Supplementary Fig. 17a). By using the zebrafish piRNA collection from piRBase Release (v3.0) (http://bigdata.ibp.ac.cn/piRBase/) as a reference database, we find that most of the small RNA reads mapped onto TE-alone loci (56.06% to 82.84%) can be annotated as piRNAs (Supplementary Fig. 17b). The remaining unannotated reads may be specific piRNA populations that have not yet been included in this database or other small RNA types. We also investigate the sequence features of these TE-related small RNA populations and identify an over-representation of uridine at the 5' most end (1U bias) and adenine at the tenth position (10A), which are other unique features of piRNAs[17,18] (Supplementary Fig. 17b). Taken together, our findings suggest that most mapped reads on active TE-alone loci belong to the piRNA species. Despite we cannot exclude that the nascent piRNAs can be generated during early embryogenesis, the piRNA-dependent TE silencing pathway is highly likely to be effective as a potential approach in post-transcriptional regulation on TE-alone transcripts.

10. Functional analysis of novel genes

To better understand the biological functions of novel genes identified during zebrafish early embryonic development, we employ weighted gene network analysis to identify gene modules exhibiting highly correlated co-expression patterns. We first identify a total of 8,738 differentially expressed genes between two adjacent stages (from the fertilized egg to shield stages), and then establish seven distinct co-expression modules that reveal diverse gene expression patterns (Fig. 6a). Furthermore, gene ontology functional analysis highlights that M1 and M2 modules have important biological functions during embryonic development (Supplementary Fig. 19a). Therefore, according to the

expression pattern, domain analysis and literature searching, we select a novel TE-gene locus, *zeat1*, and a novel regular gene, *zeat2* for further downstream experimental studies.

zeat1 has four TE-gene chimeric isoforms, with a Tc1N1_DR (an ancient nonautonomous Tc1-like DNA transposon) inserted in the last exon (Fig. 6a). However, this 679 bp insertion does not interrupt its ORF (open reading frame), therefore *zeat1* still encodes a protein with 455 amino acids. Transcript abundance of *zeat1* can be detected in the fertilized egg and 1-cell stages and decreases until the shield stage (Fig. 6b). To obtain a knockout mutant of the *zeat1* gene, we employ CRISPR-Cas9 system to modify it by targeting the first exon (Supplementary Fig. 19d). We select the mutants with a one-base pair deletion and a nine-base pair insertion for further study, which cause the frameshift in the ORF, resulting in a truncated protein losing the intact GEX1/Brambleberry domain (Supplementary Fig. 19d). We observe that zygotic *zeat1* ($zeat1^{-/-}$) mutants, obtained by crossing heterozygous male and female fish carrying $zeat1^{-/-}$ alleles, showed normal morphology during embryogenesis and could become fertile adults. Maternal *zeat1* (M*zeat1*) mutant embryos were collected by crossing $zeat1^{-/-}$ females with wild-type (WT) males (Supplementary Fig. 19c). These mutants can block the expression of *zeat1* in oocytes and further prevent maternal deposition of *zeat1* transcripts into the embryos. Prior to the mid-blastula transition (MBT, 2 hpf), M*zeat1* embryos are morphologically similar to WT embryos (Fig. 6c). However, M*zeat1* mutants fail to undergo cell movements associated with the epiboly and gastrulation, and the mutant embryos arrest development shortly after MBT, suggesting *zeat1* likely plays a critical role in maternally supported development (Fig. 6c). To further confirm whether *zeat1* is a maternal-effect gene,

maternal-zygotic *zeat1* (MZ*zeat1*) mutant embryos are also obtained by crossing homozygous male and female mutants. MZ*zeat1* mutants show similar morphology compared with M*zeat1* mutants and stop development until the shield stage (Fig. 6c), further supporting that *zeat1* is a strictly maternal-effect gene, which is essential for zebrafish early embryonic development. To test the biological functions of *zeat2*, we generate zygotic CRISPR-Cas9 mutants $zeat2^{-/-}$, which produce one mutant with one base pair deletion and the other with 5 bp deletion within the first exon, resulting in premature termination of normal translation (Supplementary Fig. 19d). $zeat2^{-/-}$ mutants develop normally, and there is no discernible difference in morphology compared to WT and heterozygous embryos in the first 3 days post fertilization (dpf) (data not shown). However, when the mutant larvae reach 8-10 dpf, they exhibit body curvature and gradually lose viability (Fig. 6f). We monitor the growth of embryos (F_2) produced from heterozygous intercrosses from 24 hours to 20 dpf. Ten embryos or larvae are randomly selected and genotyped each day. We observe that from day 18 to day 20, no homozygous mutant embryos can be detected (Fig. 6g), indicating that the homozygous mutant embryos of $zeat2^{-/-}$ cannot survive beyond day 18. Furthermore, through genotyping of over 100 F_2 adult fishes, we cannot identify any homozygous mutant individuals, suggesting that *zeat2* is a zygotic-effect, lethal gene.

11. Evaluating the impact of cytoplasmic polyadenylation during early embryogenesis.

Cytoplasmic polyadenylation has been suggested as prevalent for zebrafish maternal mRNAs during early embryogenesis[19,20]. Due to the delay of polyadenylation of maternal

mRNA, a subset of gene abundance can be underestimated with poly(A) enrichment approach.

To further confirm whether cytoplasmic polyadenylation greatly impacted the quantification of TE-alone transcripts in this study, we analyzed two publicly available RNA-seq dataset (PRJNA624126 and PRJNA529241) covering the zebrafish early development generated by both poly(A) capture and ribosomal RNA depletion library preparation methods. Theoretically, if one transcript is greatly impacted by cytoplasmic polyadenylation, the abundance estimated by poly(A) capture should be lower than ribosomal RNA depletion due to the bias of poly(A) enrichment.

For regular genes, we observed a considerable number of maternal mRNAs show quantification bias (i.e. abundance underestimation by poly(A) capture method), and the overall trend gradually declined during the onset of early development (Supplementary Fig. 20 and 21). However, for TE-alone mRNA, the bias is not significant, indicating that cytoplasmic polyadenylation may not be the important regulation as regular genes (Supplementary Fig. 20 and 21). This analysis, combined with the quite low abundance of maternal TE-alone mRNA revealed by our long-read RNA-seq data, we therefore conclude our expression landscape and corresponding trajectories should not be greatly impacted by sequencing library prepared with poly(A) enrichment method.

12. Prediction of TE-regulating TFs.

Several known transcription factors regulate TE expression and repression in mammals. For example, human cleavage-specific homeodomain transcription factor DUX4 and mouse ortholog DUX can regulate HERVL and MERVL family in human and mouse, respectively[21]. Klf5 is another transcription factor that can regulate the activation of three

ERV families in mouse 2C blastomeres[22]. In addition, Gata2, Rara Zfp281 and Tbx-family factors are also validated to regulate MERVL[22].

In terms of TE repression, the Kruppel-associated box zinc finger proteins (KRAB-ZFPs) are well known for their function in transcriptional silencing of TEs by recruiting the transcription regulator TRIM28 and H3K9me3 mediators[23]. However, KRAB-ZFPs gene family is absent from fish species, including zebrafish[24]. Recently, the Feschotte lab has reported that a large ZNF gene family can repress the TE expression during early embryonic development in zebrafish, pointing out the potential conserved approach in TE silencing[25].

To computationally predict the potential TFs in regulating TE expression or repression (Supplementary Fig. 22a), we first ran the FIMO function within the MEME Suite to search the known motifs for 550 TE loci and identified 486 TFs with potential binding sites ($P < 0.00001$) on TE's promoter regions. We then investigated the expression pattern of 2,546 annotated TFs (downloaded from AnimalTFDB 4.0) during early embryonic development in zebrafish and found that 2,120 TFs have expression ≥1 TPM. Of 486 potential TE-regulating TFs, 469 are highly expressed during early development in zebrafish, which can be separated into two major clusters (Cluster1 and Cluster2) (Supplementary Fig. 22b). Interestingly, TFs within Cluster1 are more likely to be expressed before and during ZTA, however those within Cluster2 are more likely to be expressed after ZTA or later stages. This result could serve as a starting point for further identifying TE-related TFs during zebrafish early development.

13. Transposition, not segmental duplication is the dominant mechanism in active TE loci origin

We are interested in how these 550 active TE-alone loci have evolved and survived over long-term evolution. We hypothesize that active TE transposition and segmental duplication may both contribute to the amplification of these active TE-alone loci but with distinct implications. To understand the origin of these active TE-alone loci, we establish an approach to distinguish transposition from segmental duplication by determining if the extended regions between active TE-alone loci and homologous regions are still highly identical (Supplementary Fig. 23a,b). Using 500 bp extended region as the threshold, we estimate that 82.03% of active TE-alone loci are generated by self-proliferation (Supplementary Fig. 23a). We confirm this result with various cutoff values for extended regions. With the increased length of extended regions (which means a more stringent threshold to define segmental duplication), we observe an increasing number of TE-alone loci determined as amplifying via transposition (Supplementary Fig. 23c). We find DNA transposons show a higher proportion of SD events compared to LTRs and LINEs (Supplementary Fig. 23d), likely suggesting different evolutionary selections on different types of TEs. These results suggest that even though the transposition rate is extremely low according to previous reports[26,27], transposition, not segmental duplication is the dominant mechanism under the origination of active TE-alone loci, indicating that some TE-alone loci still maintain the transposition ability and occasionally can escape from tight control of host genome. Additionally, we find that active TE-alone loci tend to cluster in genomic regions with lower recombination rates, such as chromosome 4 (sex chromosome)[28] (Supplementary Fig. 23e), potentially allowing for increased survival rate from genomic recombination elimination.

Supplementary Figures. 1-23

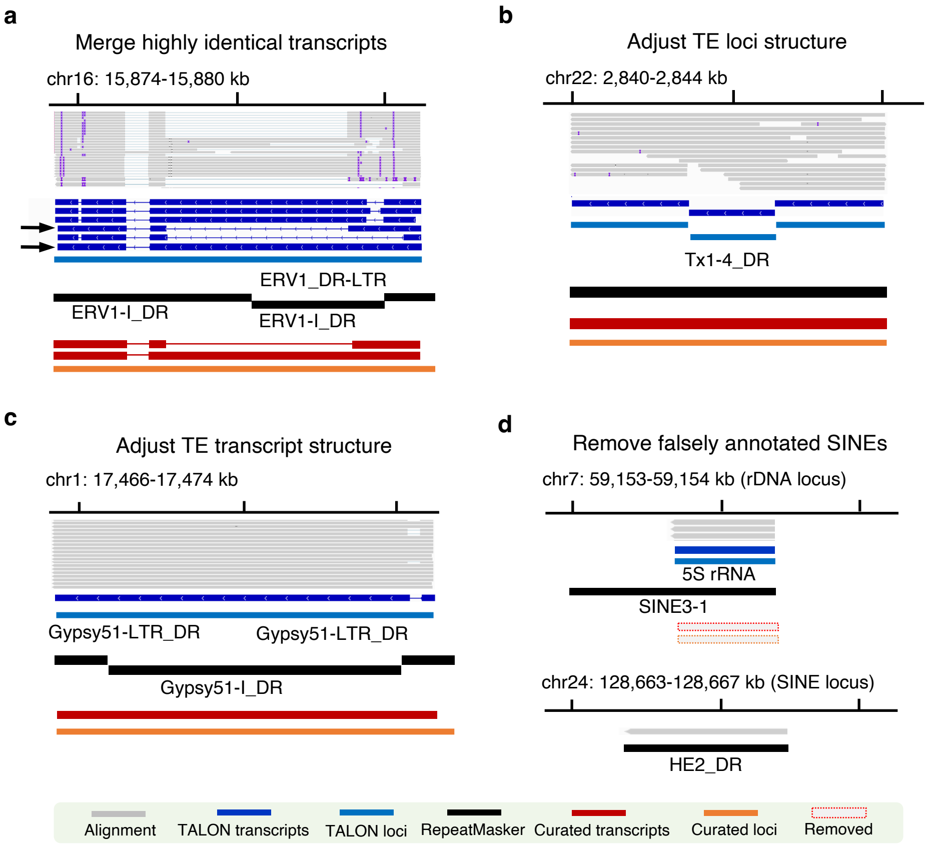

Supplementary Fig. 1 Four major issues to be solved by manual annotation in TALON-based TE-alone transcript identification. **a**, Multiple highly identical TE transcripts can be manually merged into consensus transcripts. Black arrows indicate the well-supported transcripts maintained after manual annotation. **b**, A single TE-alone locus is identified as multiple fragmented TE-alone loci that can be manually integrated/combined into single one. **c**, Manually correct the mis-annotated introns caused by short deletions during alignment. The intron gap can be filled to get an intronless structure. **d**, 5S rDNA loci can be incorrectly annotated as SINE loci due to the sequence similarity between SINE3-1 and rDNA (upper panel). HE2_DR represents a real SINE locus (lower panel). Alignment, long read alignment by minimap2; RepeatMasker, TE annotation; TALON transcripts and loci, generated by TALON; Curated transcripts and loci, generated by manual curation.

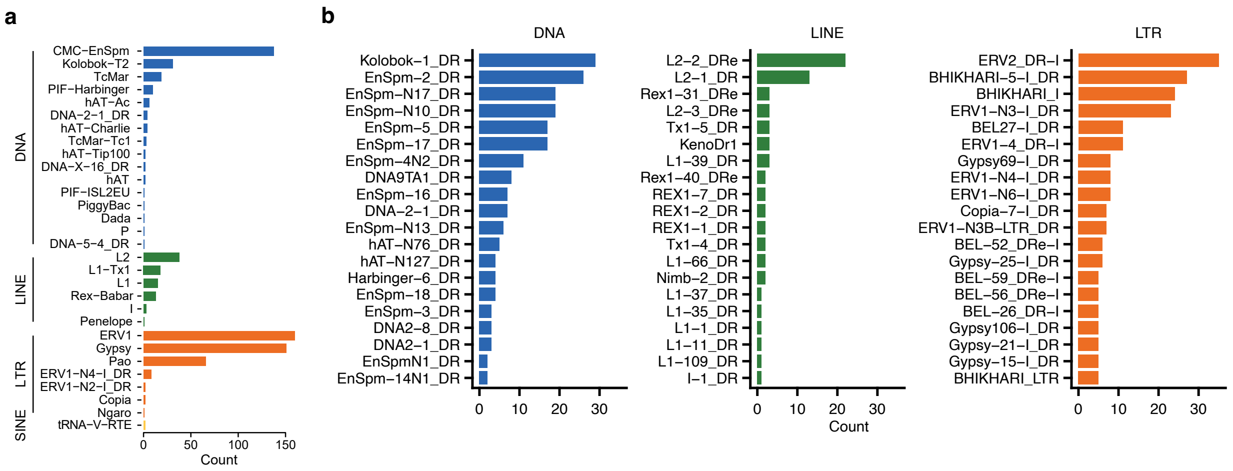

Supplementary Fig. 2 Classification of TE-alone transcripts. **a**, TE family classification. **b**, TE subfamilies classification. The top 20 subfamilies with the most transcripts are listed for each TE type. The X-axis represents the number of TE-alone transcripts in each family/subfamily.

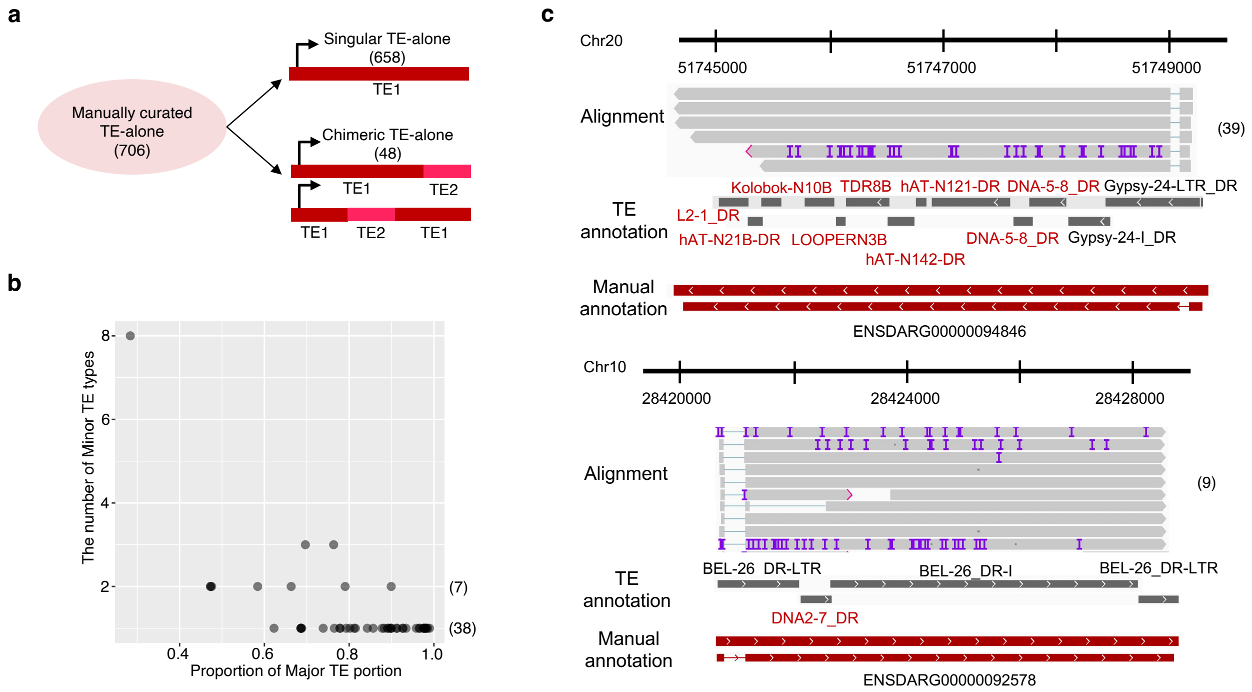

Supplementary Fig. 3 Characterization of chimeric TE-alone transcripts. a, TE-alone transcripts can be divided into singular (transcribed from one single TE loci) and chimeric (comprising of multiple TE fragments from different subfamilies) TE-alone transcripts. TE1 and TE2 represent TE copies from different TE subfamilies. **b**, Component analysis of chimeric transcripts. A scatter plot is drawn with the sequence proportion of the major TE portion (x-axis) and the number of minor TE subfamilies (y-axis). 38 and 7 represent the number of transcripts (dots) containing 1 or 2 kinds of minor TEs, respectively. Few chimeric TE-alone transcripts have a more complicated structure. **c**, Chimeric TE-alone transcripts can originate from readthrough transcription (upper panel) and nested TE insertion (lower panel). Two examples to depict the two approaches of chimeric TE-alone transcripts formation. Of 48 chimeric transcripts, 39 are classified as readthrough transcription and 9 as nested TE insertion.

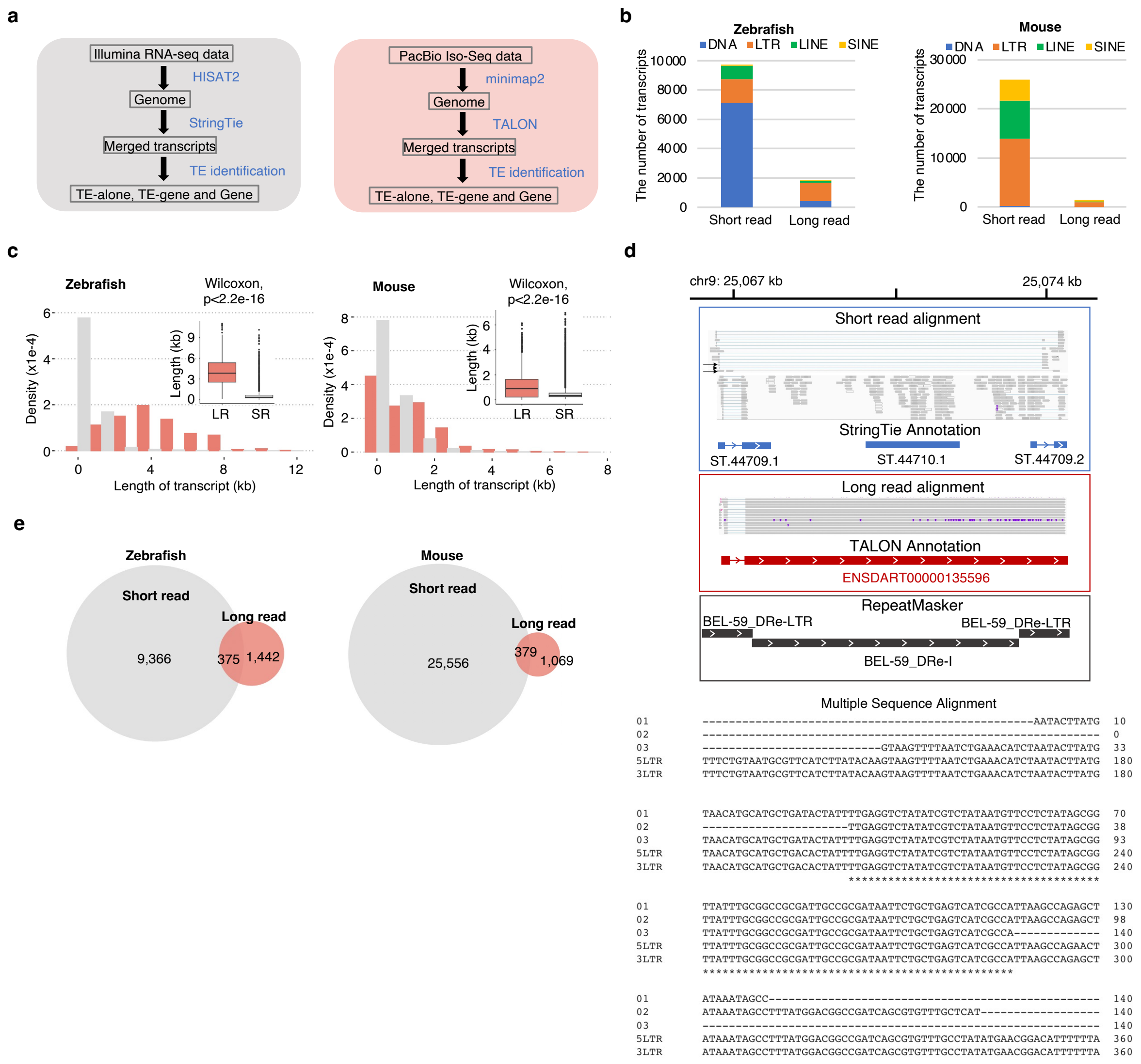

Supplementary Fig. 4 Comparison of TE-alone transcript identification between short-read and long-read RNA sequencing technologies. a, Pipelines for TE-alone transcript identification with Illumina short-read RNA-seq data and PacBio Iso-Seq data. **b**, Comparison of the number of TE-alone transcripts identified by short-read and long-read RNA-seq data in zebrafish and mouse. **c**, Length distribution of transcripts identified by short-read (SR) and long-read (LR) RNA-seq data. Wilcoxon rank sum tests, two-sided are applied. $P < 2.2 \times 10^{-16}$ indicates an extreme small p value. **d**, Genome browser snapshot showing an example of the different transcript identification results on the same TE-alone locus. Long-read data annotates this region as a single LTR locus, while short-read data characterizes three loci due to the noncontinuous alignments. Three black arrows indicate three alignments from three reads with identical sequences, which can be either mapped on one 5'LTR or split into two segments and mapped onto 5'LTR and 3'LTR, respectively, due to the similar sequences of two LTRs. Multiple sequence alignment between three reads and two LTR sequences show the high sequence identity. **e**, Number of TE-alone loci shared by both short-read and long-read data in zebrafish and mouse.

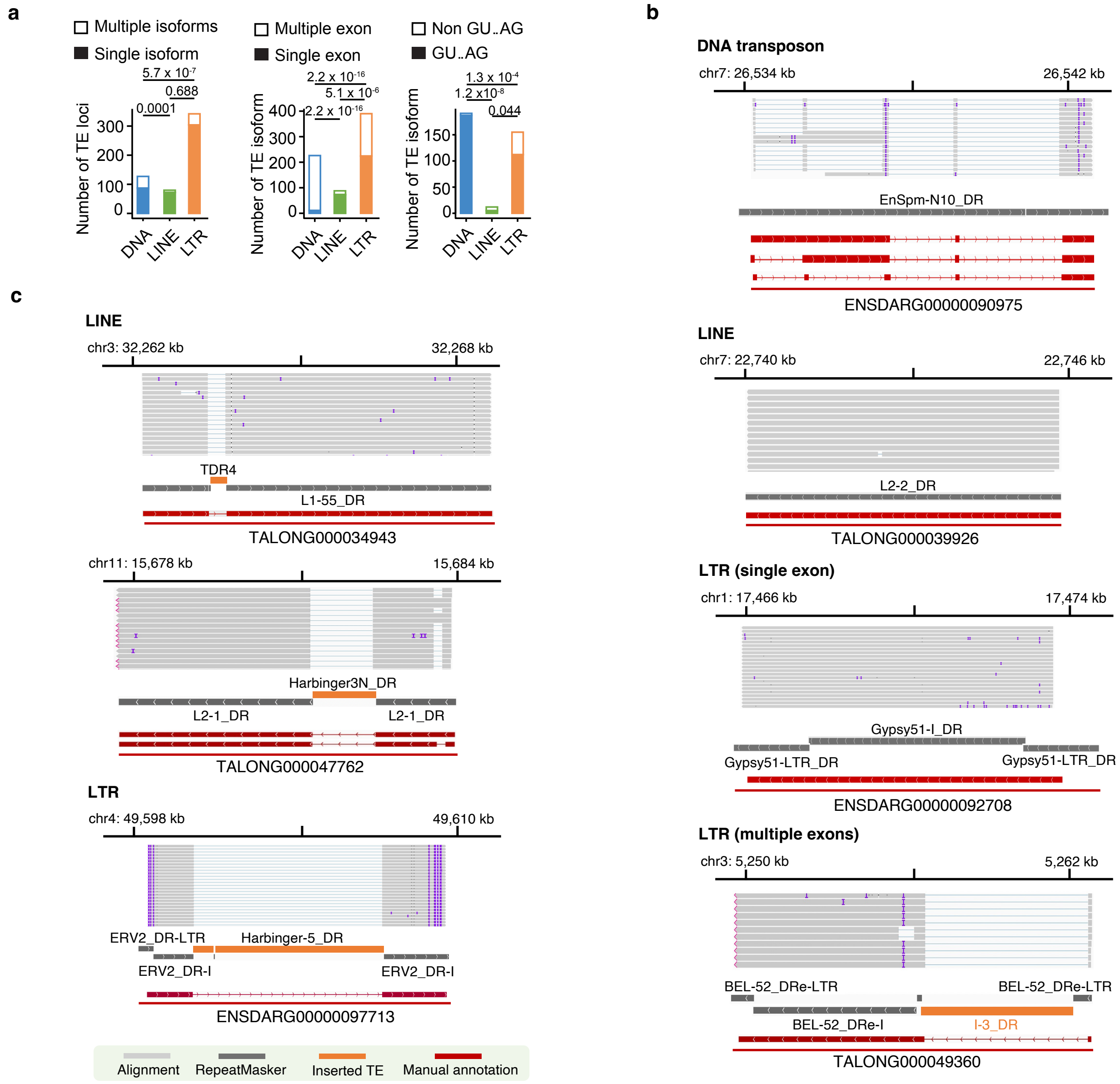

Supplementary Fig. 5 Transcription patterns of DNA transposon, LINE and LTR. a, Difference in isoform number, exon number, and splicing signal, for each TE-alone transcript/locus among DNA transposons, LINEs, and LTRs. Fisher's exact test was used to determine the statistical significance. **b**, Examples to show typical transcription pattern of different types of TEs. DNA transposon often has multiple transcript isoforms and undergoes alternative splicing. LINE often undergoes "head-to-tail" mode transcription without introns. Some LTRs show similar transcription as DNA transposon; other perform similar transcription as LINE. **c**, Spliced introns can be originated from TE insertion. When transcription occurs, the inserted TE elements are spliced as introns.

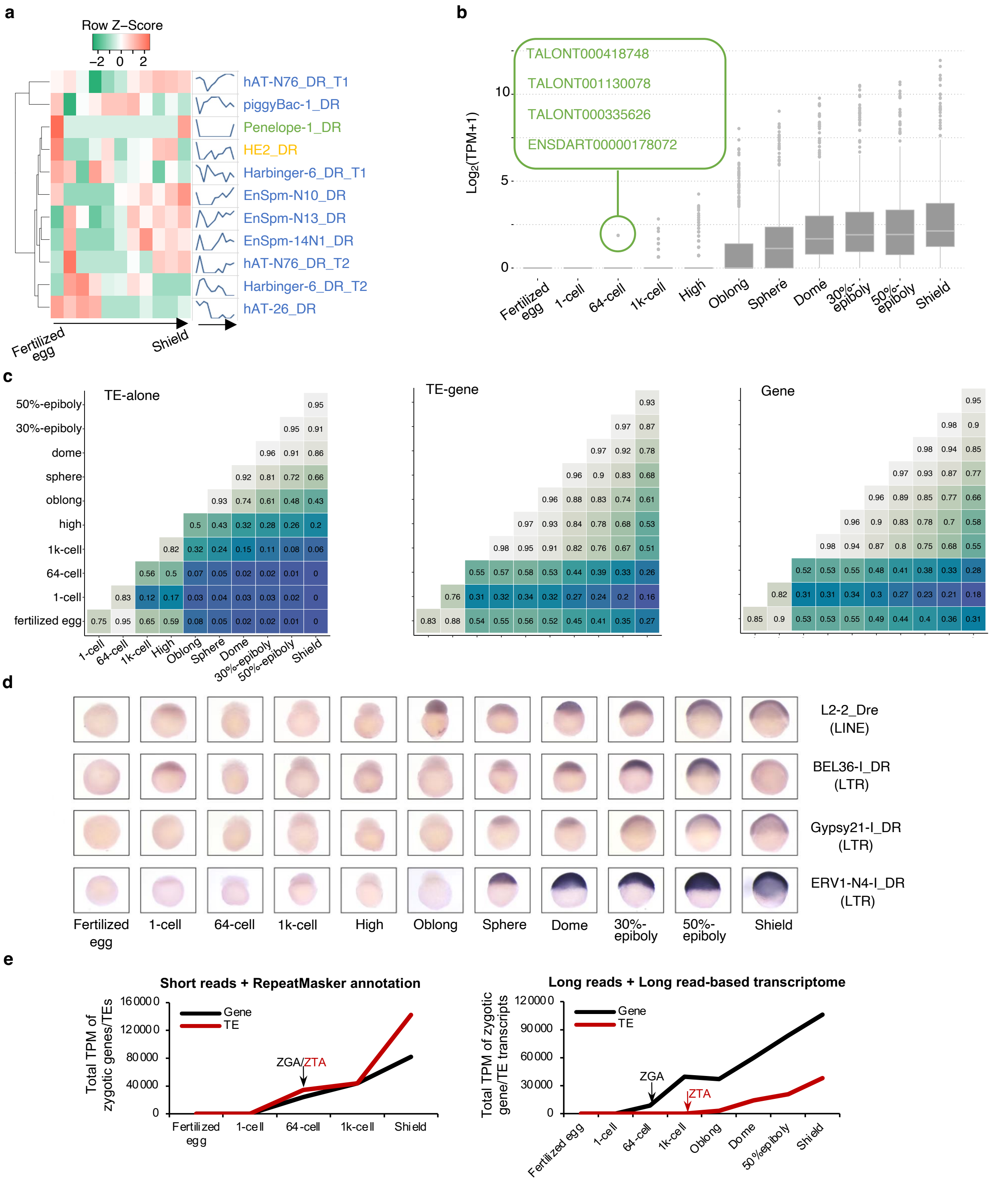

Supplementary Fig. 6 Maternal inherence and zygotic activation of TE-alone transcripts. a, Expression pattern of 11 maternally inherited TE-alone transcripts, including nine DNA transposons (blue color), one LINE (green) and one SINE (yellow). **b**, Boxplot showing expression levels of zygotically activated TE-alone transcripts. Four TE-alone transcripts, detected at the 64-cell stage with only one supported read, are

highlighted with their corresponding transcript IDs. The boxplots show the center as median, lower bound of the box as the first quartile (Q1), upper bound of the box as the third quartile (Q3) and lower whisker (Q1 – 1.5 x IQR) and upper whisker (Q3 + 1.5 IQR) as minima and maxima. **c**, Pearson's correlation coefficients of stage-by-stage expression for TE-alone, TE-gene and gene groups. **d**, WISH experiments showing the expression of four TE subfamilies across 11 developmental stages. **e**, Define the initial stages of ZGA and ZTA with short-read and long-read RNA-seq data (see Methods).

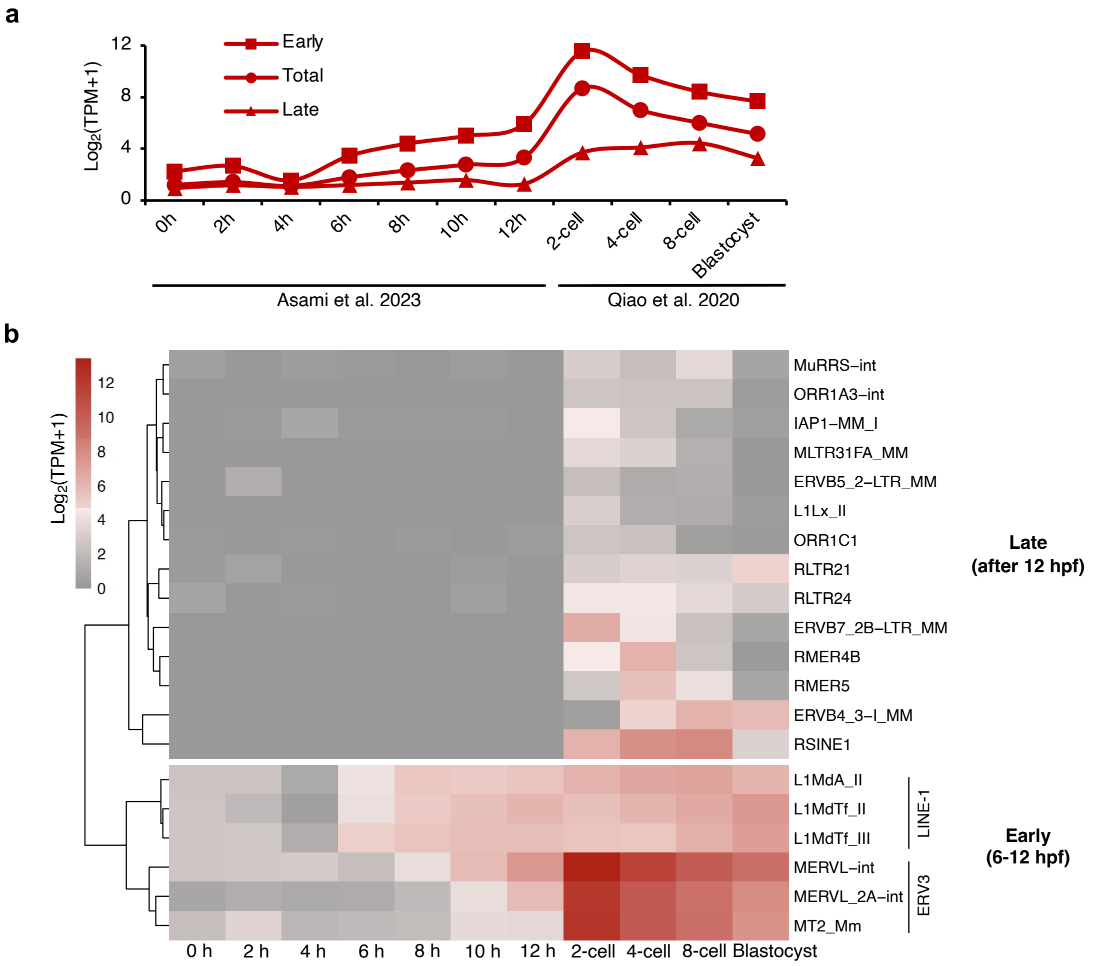

Supplementary Fig. 7 Activation of TE subfamilies during early mouse embryonic development. a, TE subfamilies activated during the early stages of mouse embryonic development. The TE subfamilies (Total) displaying upregulation before 12 hours post fertilization (hpf) were classified as the early activation group (Early), while those upregulated after 12 hpf were categorized as the late activation group (Late). Data sources are listed at the bottom. **b**, The heatmap illustrates the activation patterns of the selected mouse TE subfamilies. Most subfamilies become activated after 12 hpf, except for three LINE-1 subfamilies showing upregulation at 6 hpf, and three LTR subfamilies being activated at 8 hpf or later.

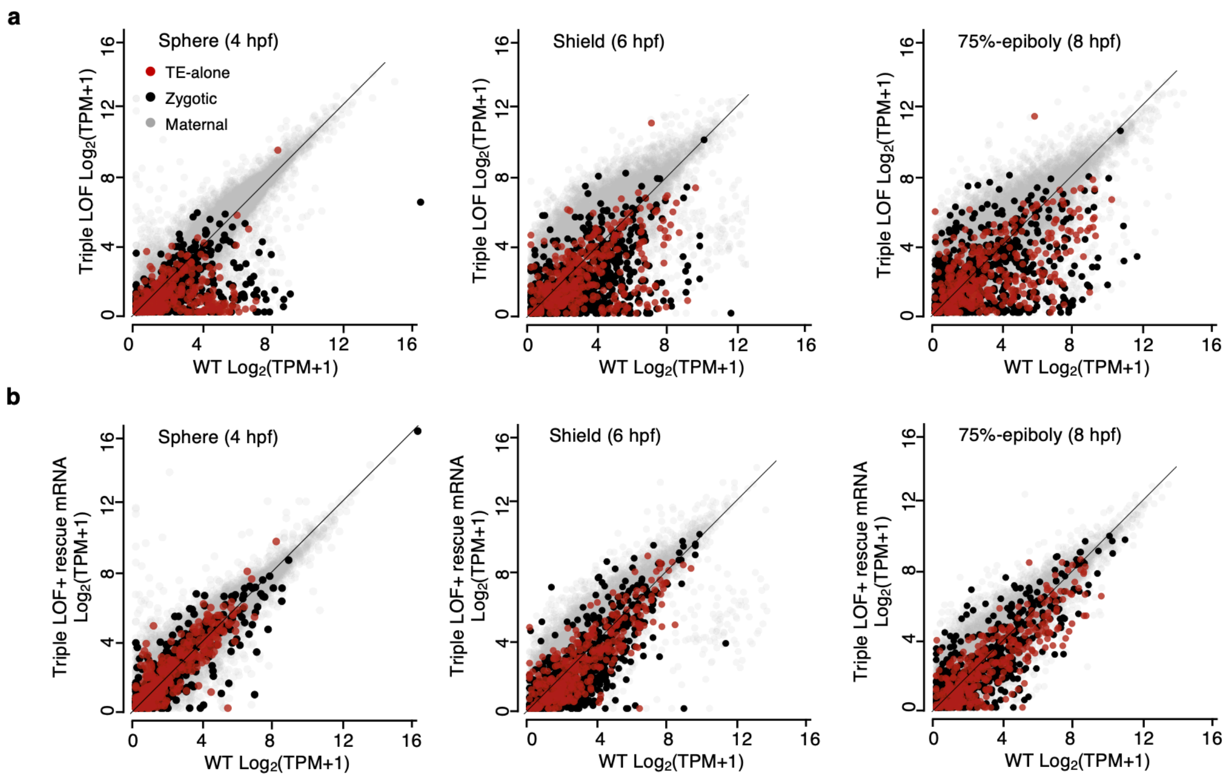

Supplementary Fig. 8 Key transcription factors impact ZTA. a, Scatter plots showing the widespread expression loss of TE-alone loci and genes in triple LOF mutant at 4, 6 and 8 hpf. TE-alone loci and gene expression loss in triple LOF mutants of maternal zygotic *pou5f1* (MZ*pou5f1*) with Nanog and SoxB1 morpholinos co-injection. **b**, mRNA rescue can restore normal ZTA. Injection of *pou5f1*, *nanog* and *soxB1* mRNAs at the 1-cell stage can rescue both the expression of TE-alone loci and genes.

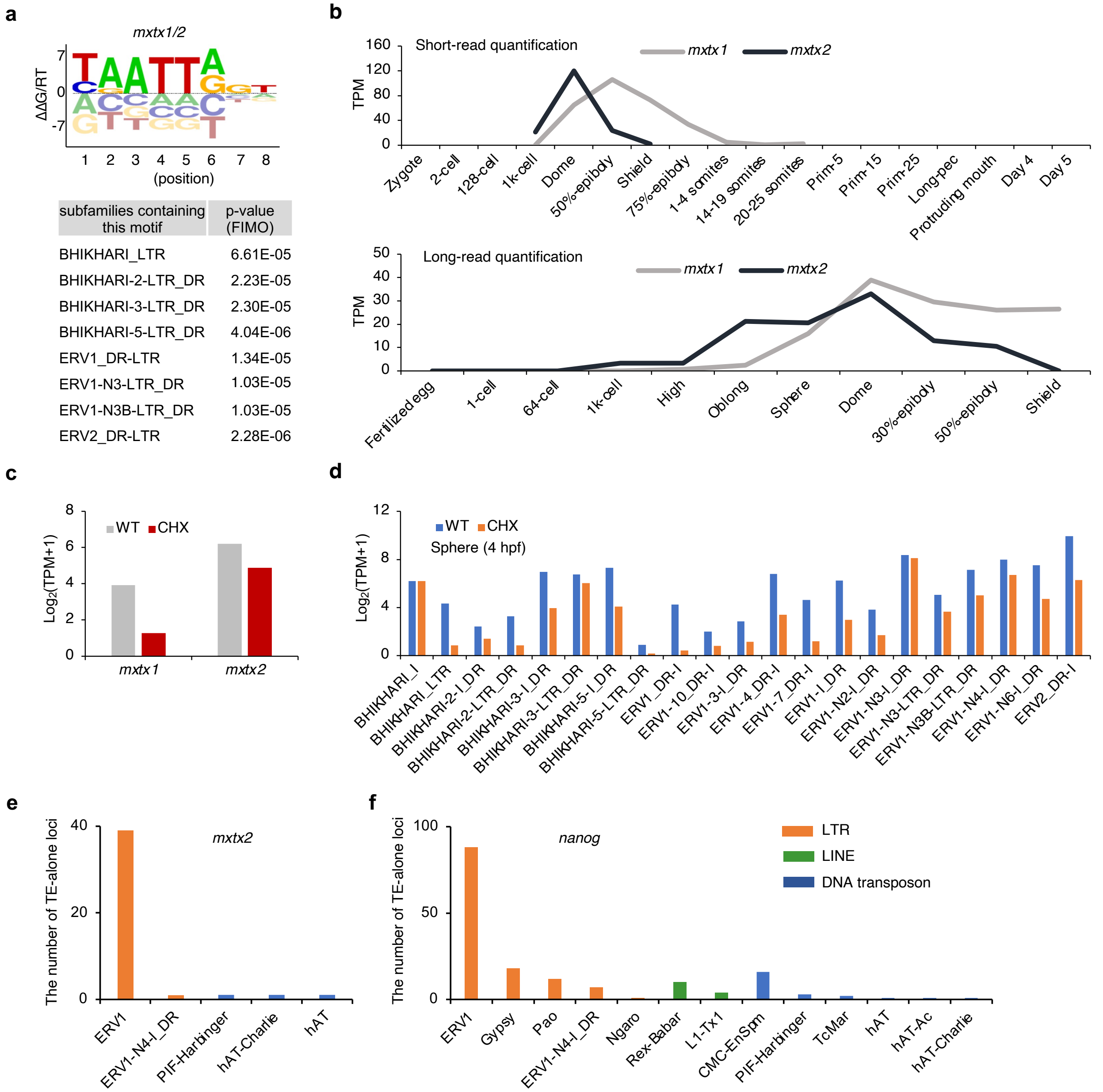

Supplementary Fig. 9 Analysis on regulatory roles of *mxtx1/2* on TE activation. a, Predicted binding motifs of *mxtx1/2* and TE subfamilies (from ERV families) containing this predicted motif. **b**, Expression pattern of *mxtx1/2* characterized by both short-read and long-read RNA-seq data. *mxtx1/2* are initiated from the high to oblong stage or from the 64-cell to 1k-cell stages, respectively. *mxtx1/2* are silenced at the 14-19 somite stage or the shield stage, respectively. **c**, Despite without direct measurement of protein levels of *mxtx1/2* between wild type and CHX treatment, the gene expression levels were found to be lower after CHX treatment. This expression reduction is supposed to be mainly caused by the blockage of Nanog protein translation, which directly activates *mxtx2*[29]. **d**, Downregulated zygotic activation in zebrafish ERV subfamilies at the sphere stage following CHX treatments. **e**, Mxtx2 can specifically bind the ERV subfamilies. **f**, Nanog can generally bind to a broader spectrum of TE subfamilies.

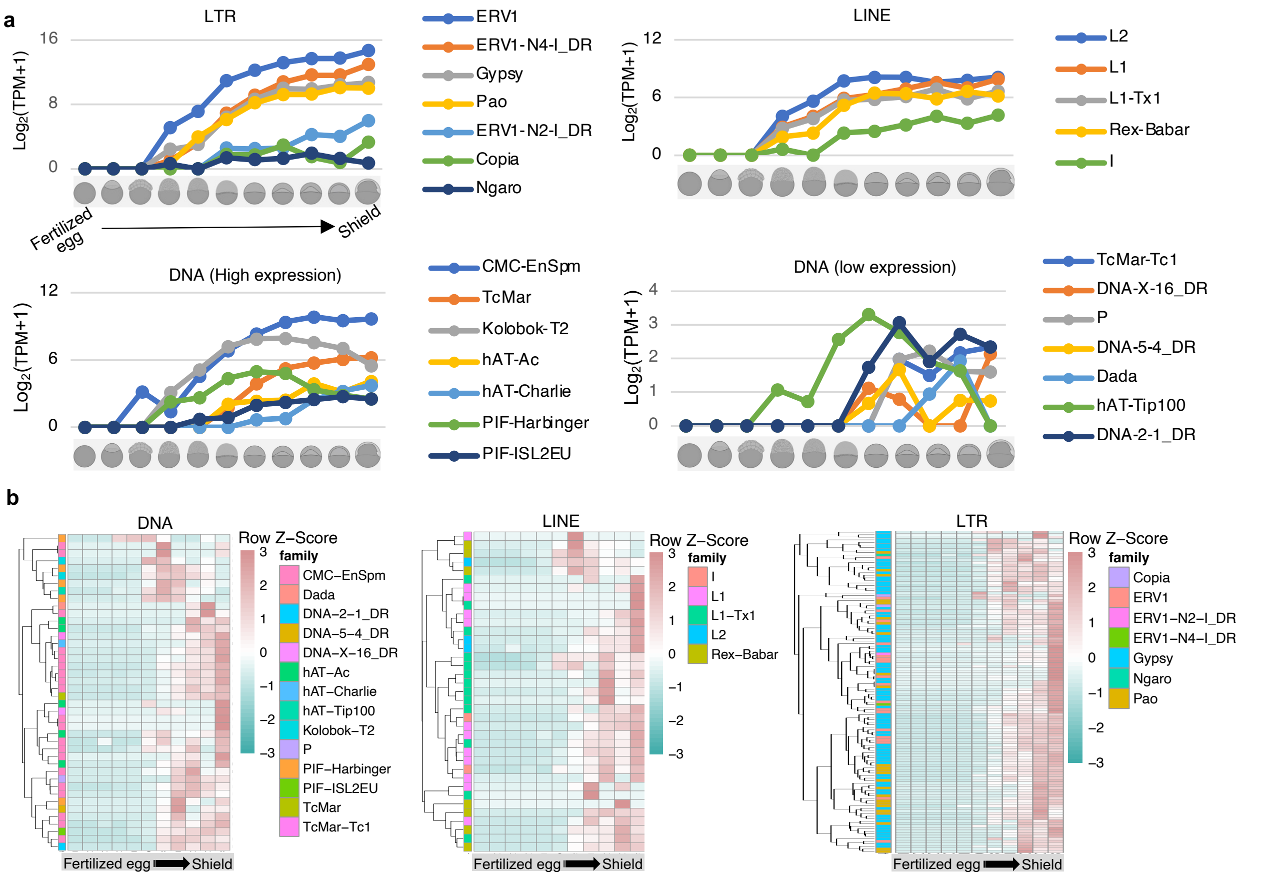

Supplementary Fig. 10 Expression patterns of TE-alone transcripts on the family and subfamily levels. a, Expression patterns of TE-alone transcripts for LTRs, LINEs and DNA transposons at the family level. DNA transposons, which have too many different families, are divided into two groups based on expression levels. **b**, Heatmaps showing the expression TE-alone transcripts at the subfamily level. SINE and maternally deposited transcripts are excluded from this analysis.

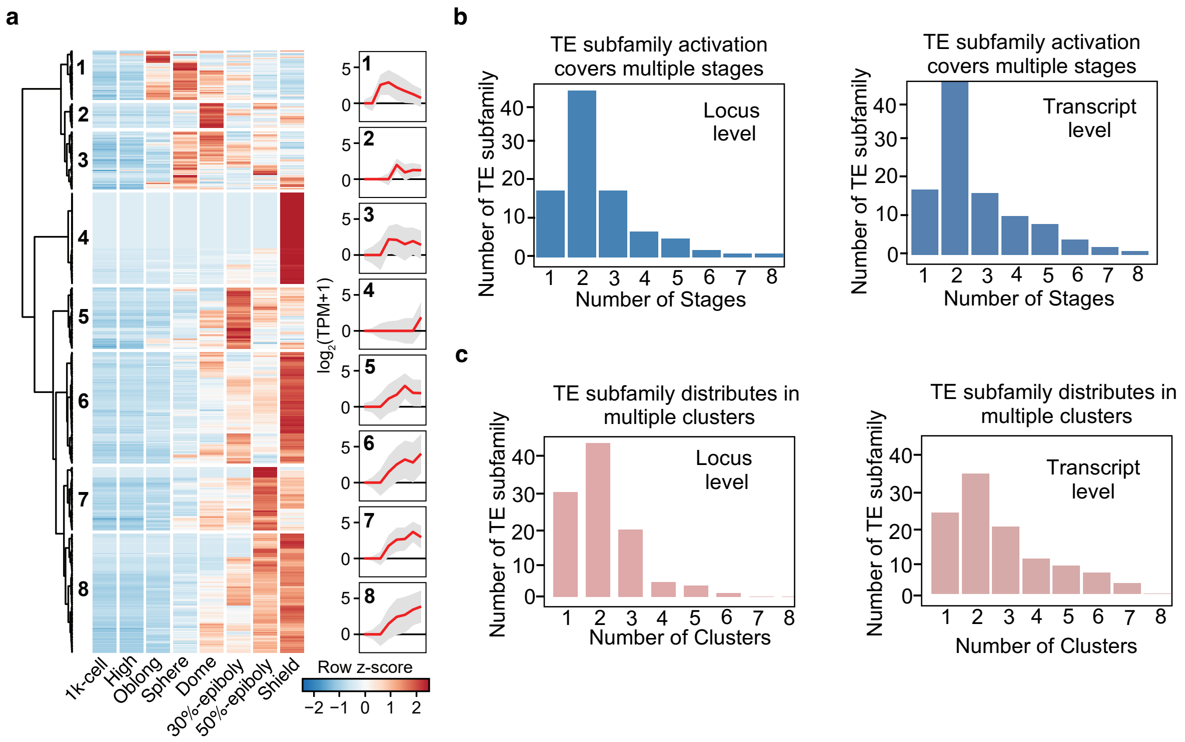

Supplementary Fig. 11 Locus-level expression analysis on TE-alone loci. a, Expression pattern for all zygotically activated TE-alone loci. Eight major clusters are established. **b**, Activation stages summary for multiple-copy TE-alone subfamilies (locus and transcript level). Bar plot showing the number of subfamilies covering different numbers of activated stages. **c**, Expression cluster summary for multiple-copy TE-alone subfamilies (locus and transcript level). Bar plot showing the number of subfamilies covering different numbers of clusters. Eight developmental stages from the 1k-cell to shield stages are counted.

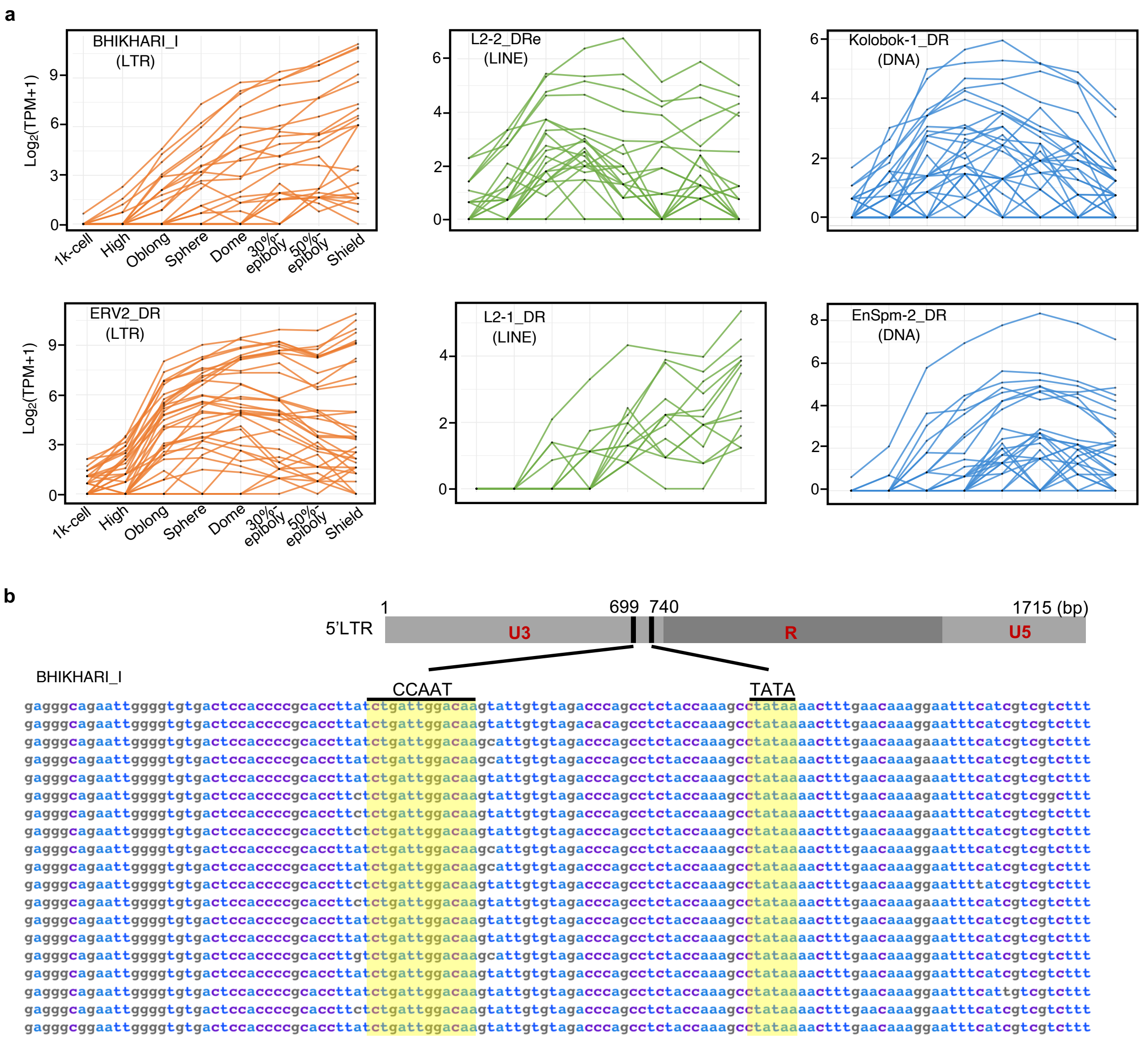

Supplementary Fig. 12 Expression heterogeneity among TE-alone transcripts within subfamily. a, Expression pattern for transcripts within TE subfamilies. Two examples were selected for LTRs, LINEs and DNA transposons, respectively. **b**, Sequence analysis of the regulatory region of the BHIKHARI_I subfamily. Multiple sequence alignment with the 5'LTR regions of 19 loci from the BHIKHARI_I subfamily shows high sequence similarity, including two conserved promoter motifs (CCAAT and TATA as highlighted in yellow).

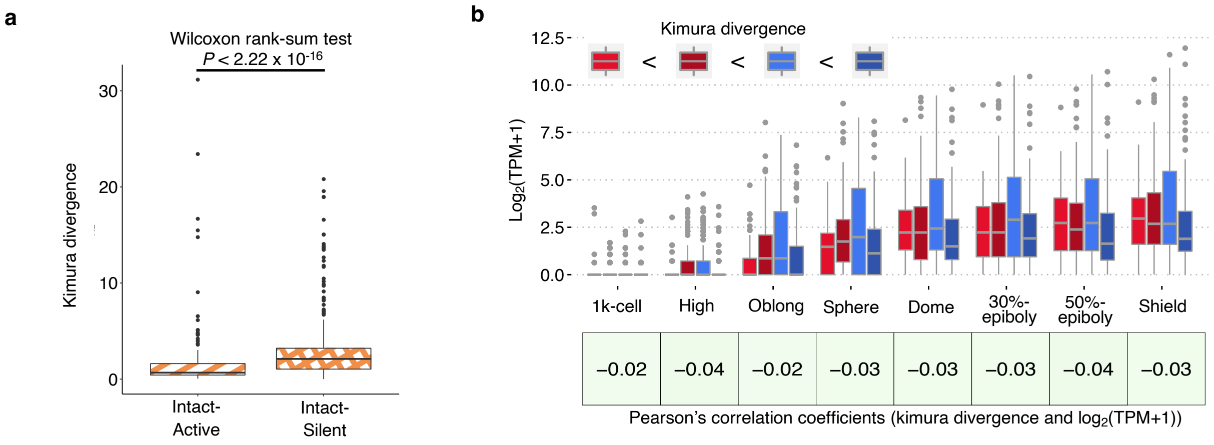

Supplementary Fig. 13 Kimura divergence analysis. a, Kimura divergence (KD) between intact LTRs that are active or silent. Wilcoxon rank sum tests, two-sided was applied to compare the significance. **b**, Correlation analysis between KD and expression at eight developmental stages. Boxplot showing the expression patterns of four subgroups of TE-alone loci separated by KD values. The four subgroups are colored from bright red to dark blue according to the increasing KD values. The table below shows the Pearson's correlation coefficients between KD and expression at each stage.

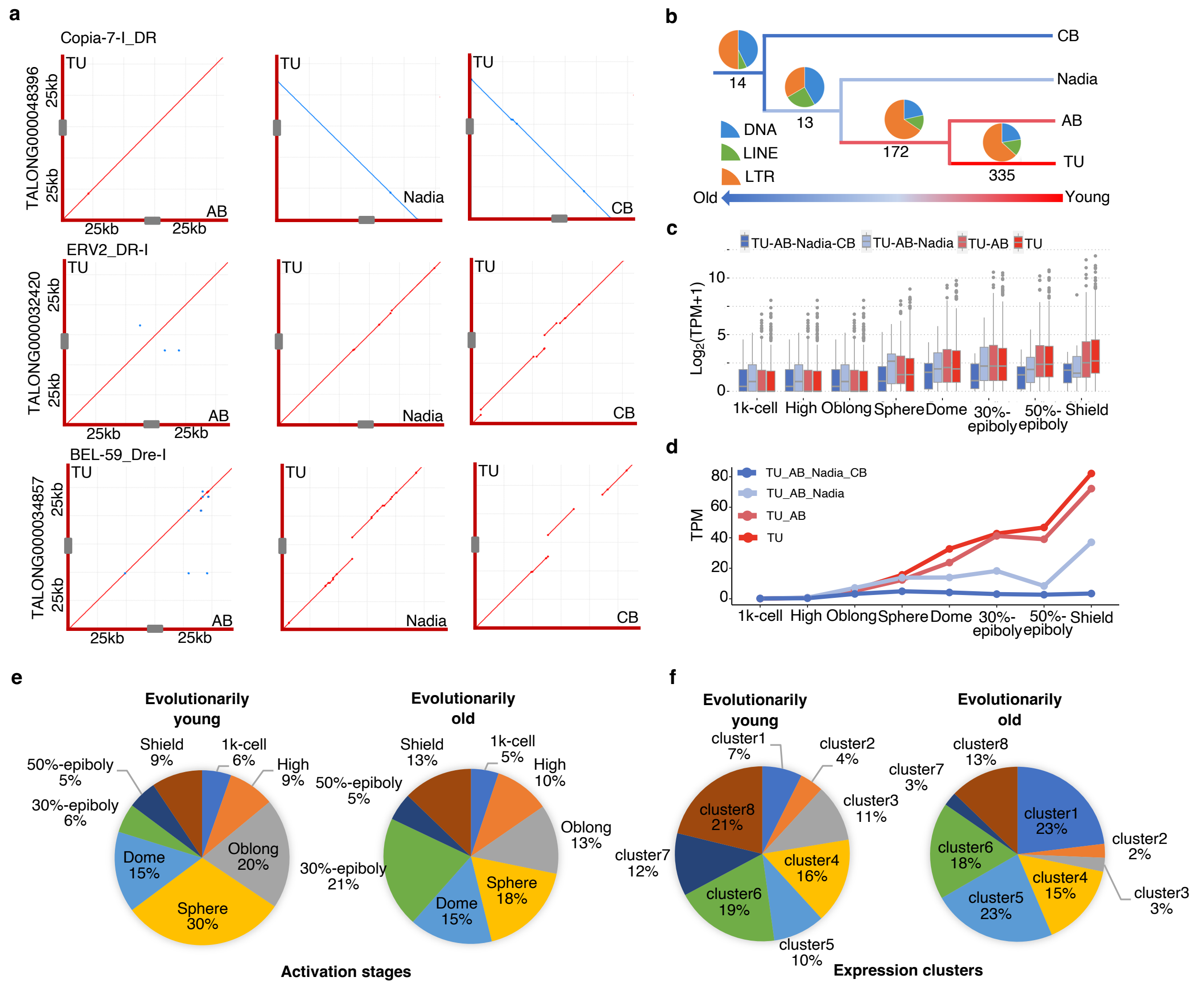

Supplementary Fig. 14 Comparative genomic analysis to infer evolutionary ages of TE. a, Comparative analysis to infer the presence and absence of active TE-alone loci in other zebrafish strains. Three examples show different types of TE collinearity: presence in all four genomes (top); presence in TU, AB and Nadia, but absence in CB (middle); and presence in TU and AB, but absence in Nadia and CB (bottom). Blue dots indicate sequence inversion. **b**, Summary of TE-alone loci across the evolutionary pedigree of four zebrafish strains. TU-specific TEs are defined as youngest and the TEs shared by all strains are the oldest. Pie charts show the proportion of DNA transposons, LTRs and LINEs for a certain group. **c**, Boxplot showing the expression of each evolutionary group over embryonic development. **d**, Line plot showing the expression tendency of each group using the mean TPM values. **e**, The proportion of TE-alone loci in different activation stages for both evolutionarily young and old groups. **f**, The proportion of TE-alone loci in different expression clusters (from Supplementary Fig. 11a) for both evolutionarily young and old groups.

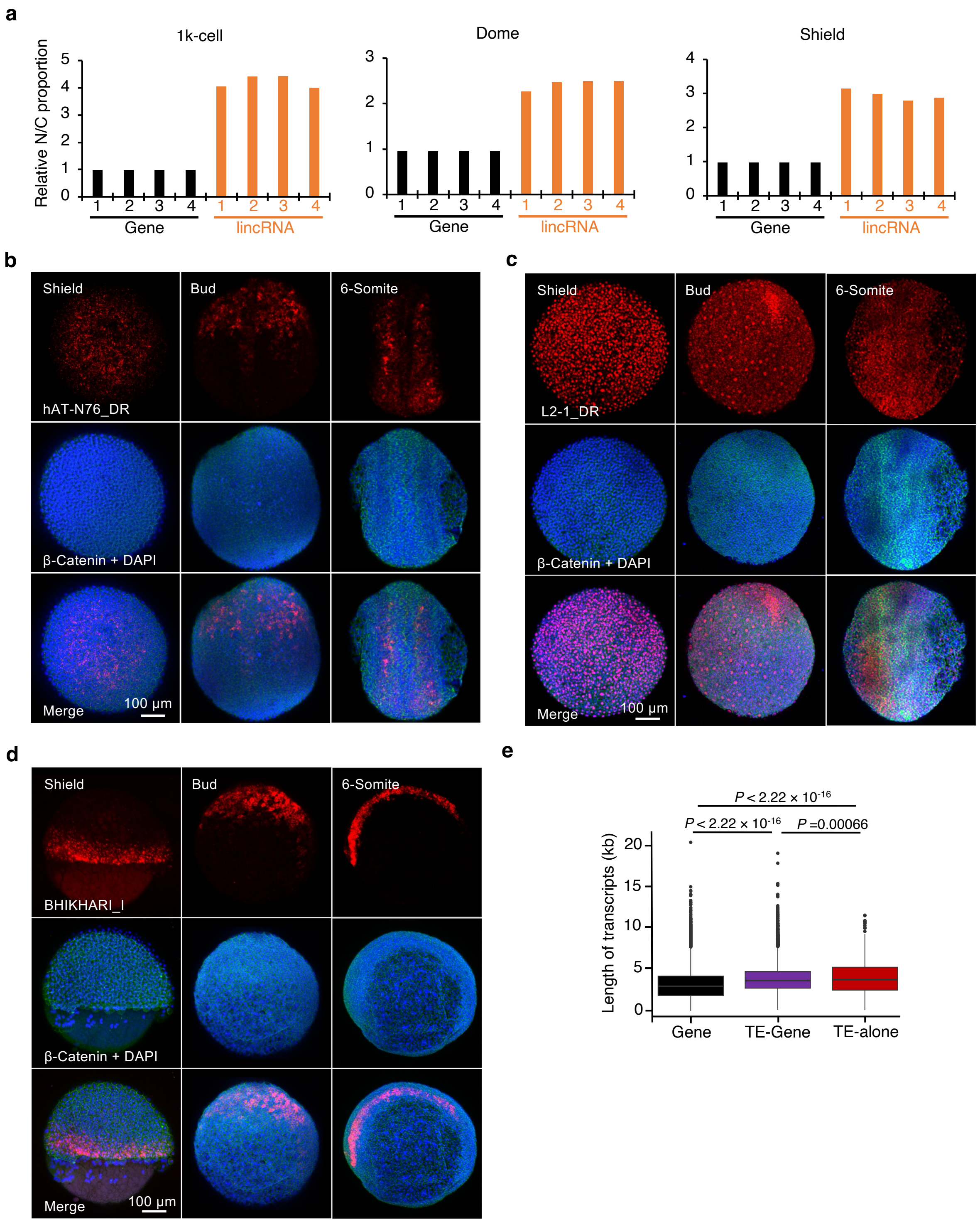

Supplementary Fig. 15 Subcellular localization. a, Relative N/C proportion between zygotically activated protein-coding genes and lincRNA (Ensembl annotation) at the 1k-cell, dome and shield stages. The results from four replicates of RNA-seq data were shown. **b-d,** FISH experiments showing the distribution of transcripts from the three selected TE subfamilies at indicated stages. Green, β-Catenin staining for cell membrane;

Blue, DAPI staining for nucleus; Red, specific probes against indicated TE-alone transcripts. Scale bar = 100 μm. **e**, Comparison of transcript length among gene, TE-gene and TE-alone groups. Wilcoxon rank sum tests, two-sided were applied to test the significance. $P < 2.2 \times 10^{-16}$ indicates an extreme small p value.

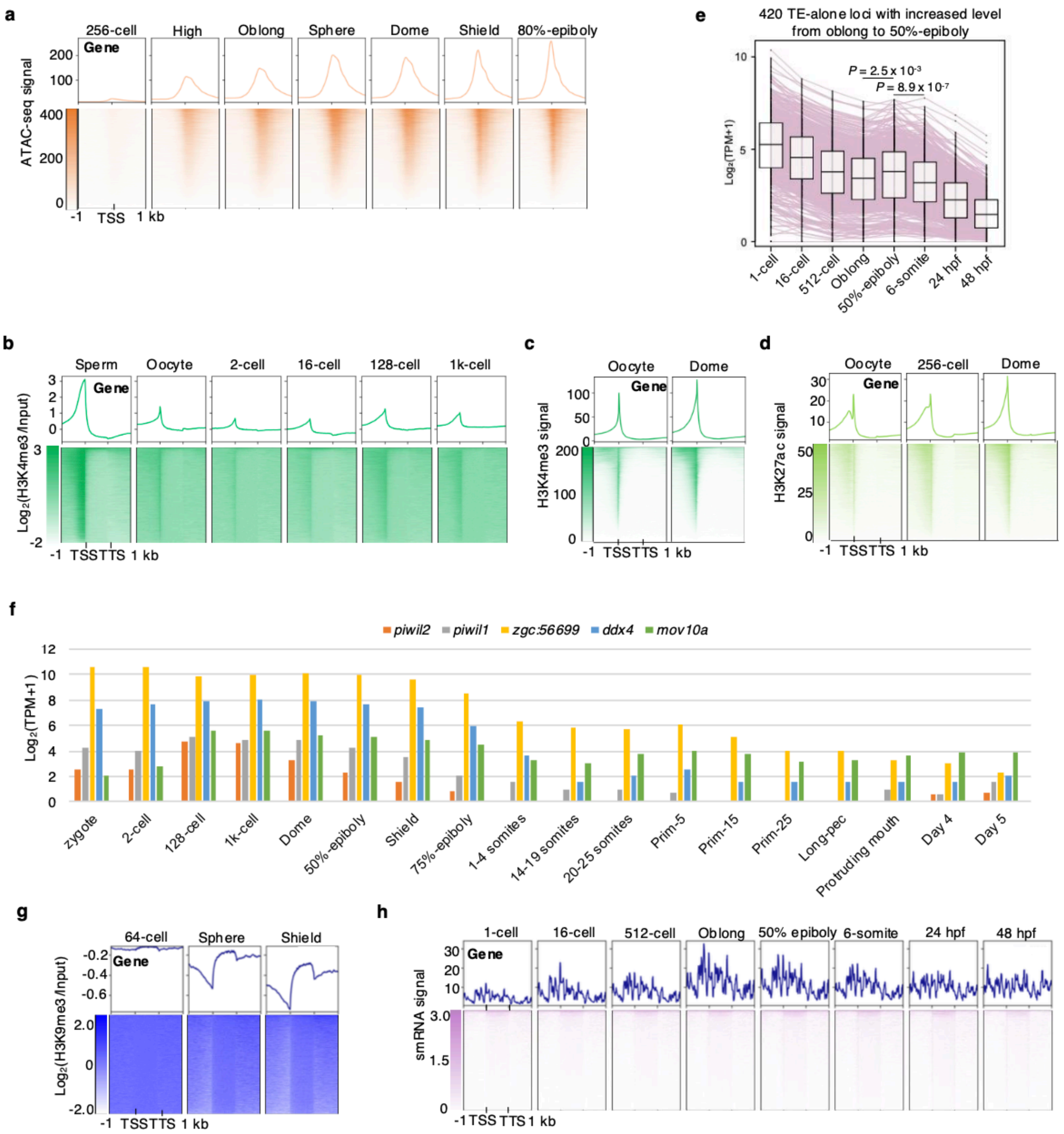

Supplementary Fig. 16 Epigenetic analysis. a, Chromatin accessibility profile for gene loci. **b** and **c**, H3K4me3 histone modification across gene regions. **d**, H3K27ac histone modification across gene regions. **e,** Expression dynamics of small RNAs mapped onto TE-alone loci, with a significant inflection at the 50%-epiboly stage. Wilcoxon rank-sum test was applied. **f**, Expression patterns for genes related to PIWI-piRNA silencing pathway over early development. **g**, H3K9me3 histone modification across gene regions. **h**, Small-RNA profile across gene loci. TSS, transcription start sites. TTS, transcription termination sites.

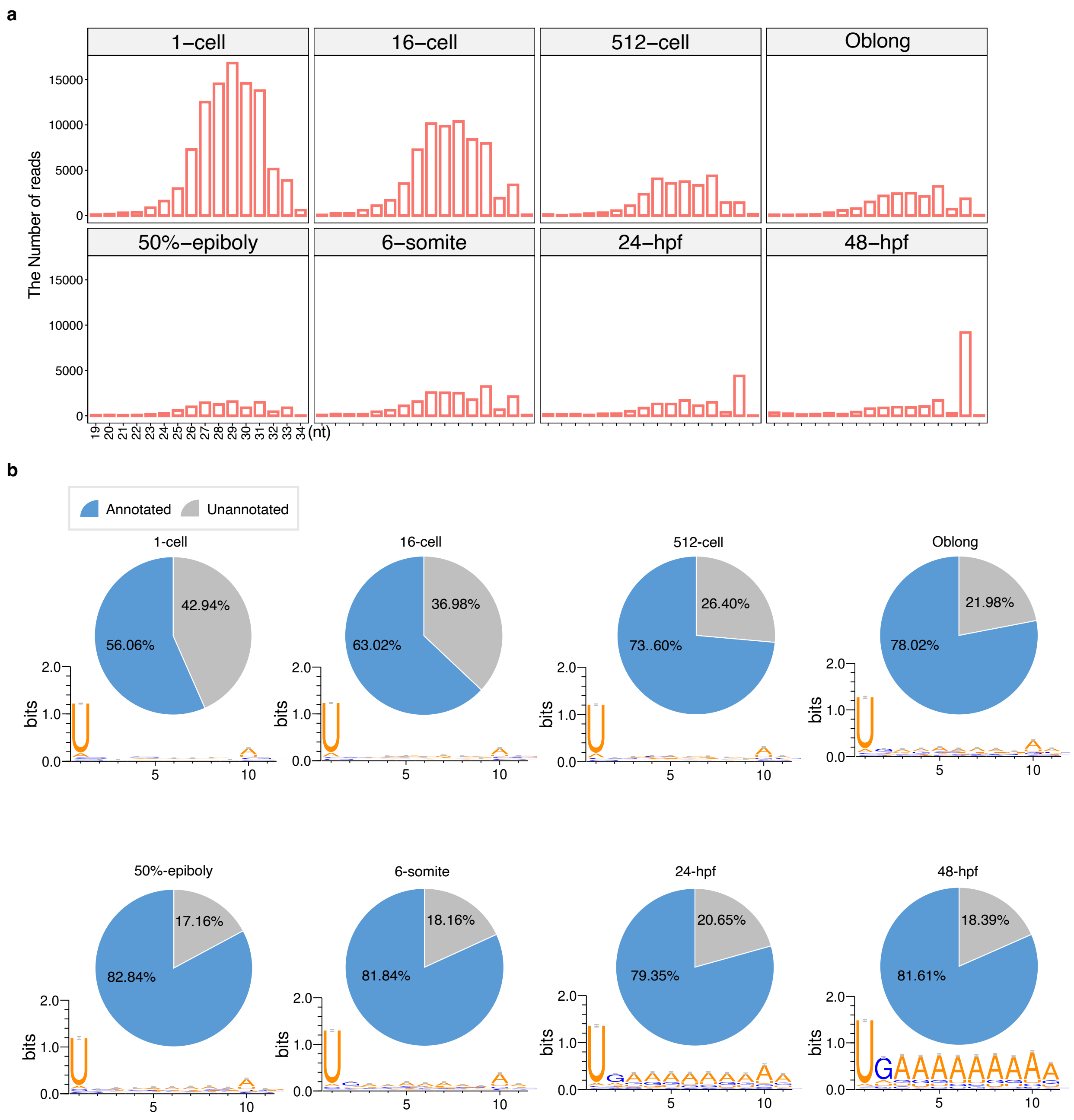

Supplementary Fig. 17 piRNA annotation and sequence feature analysis. a, Read length distribution for small RNA reads aligned onto TE-alone loci. **b**, Pie charts showing the percentage of small RNA reads that can be annotated as zebrafish known piRNAs (blue) or not (grey). A sequence logo is used to illustrate the nucleotide representation from the 1st to the 11th position for small RNA reads mapped onto TE-alone loci.

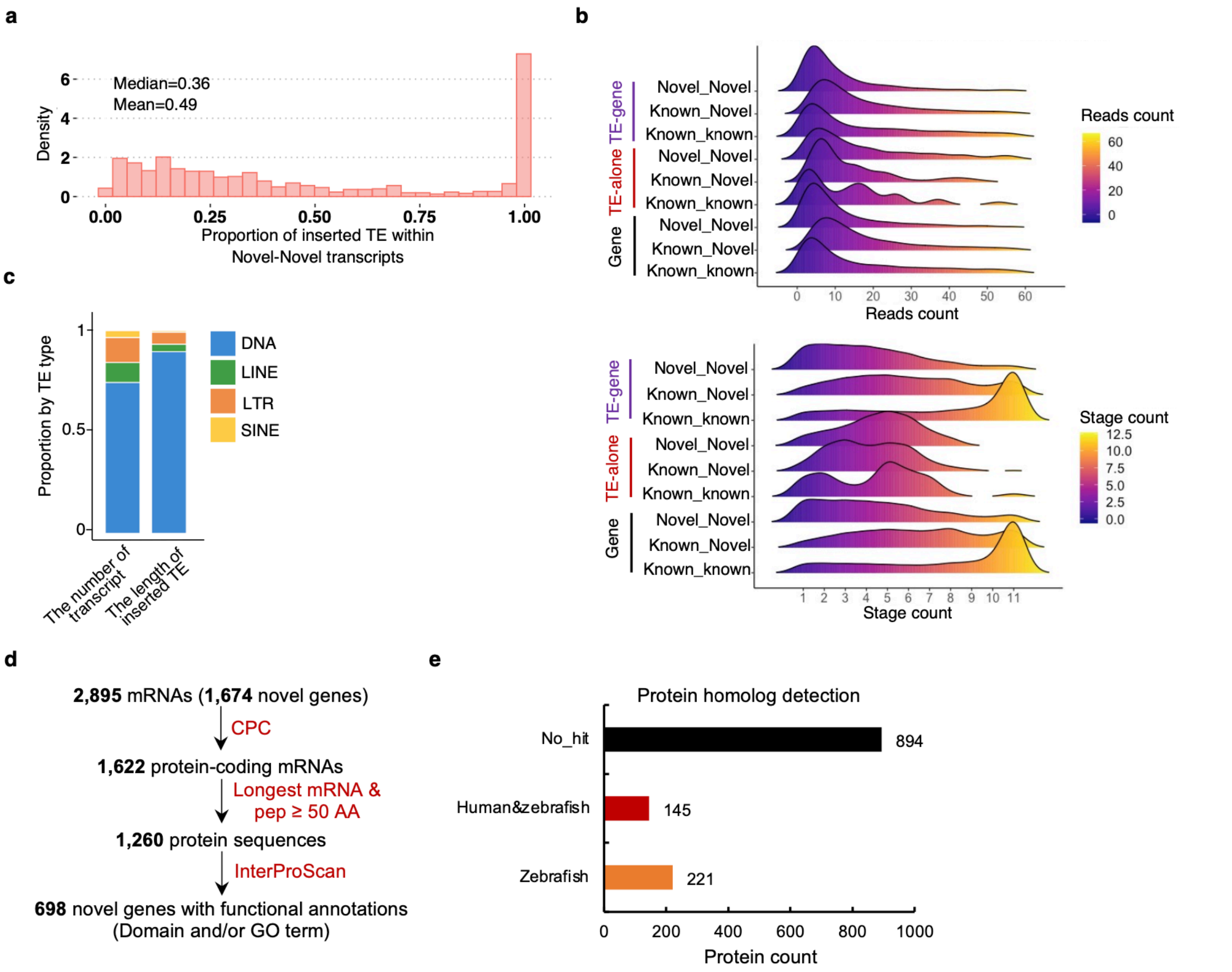

Supplementary Fig. 18 TE-gene chimeric transcripts. a, Proportion of TE sequences inserted in TE-derived novel transcripts. The large proportion of repetitive elements can greatly impact their gene annotation. **b**, Supporting evidence for identified transcripts. The number of CCS reads to support the transcript identification (read count > 60 are not shown) (top) and the number of stages where the transcript can be detected (as biological replicates) (bottom). Of note, the annotated transcripts from annotated loci (Known_known) tend to express at all 11 developmental stages, while novel transcripts tend to be stage-specifically expressed. **c**, Proportion of different types of TE sequences inserted within TE-gene transcripts. **d**, The workflow for the functional analysis of identified unannotated gene loci (both gene and TE-gene). **e**, Alignment results of the protein sequences of novel genes against the known human and zebrafish protein sequences.

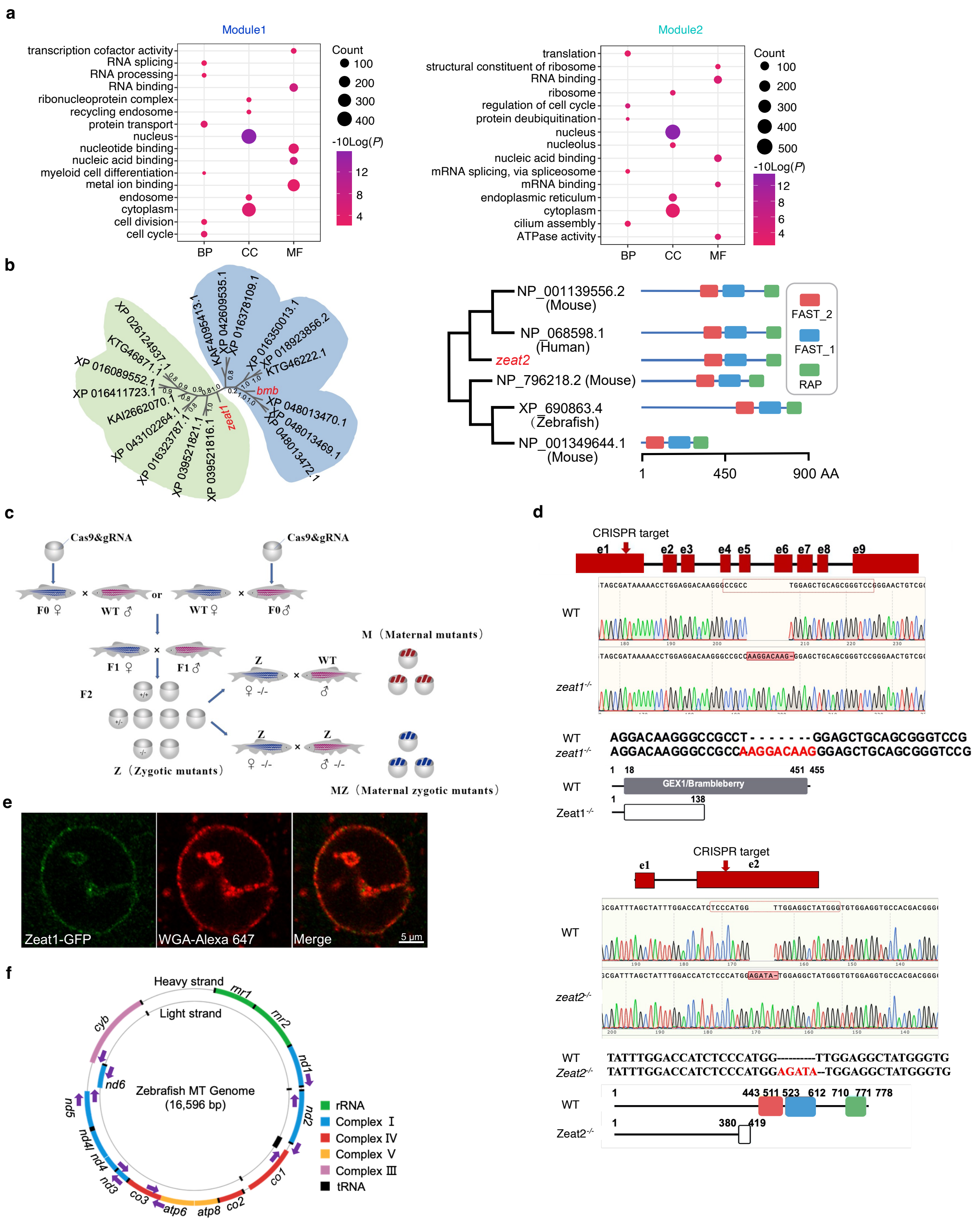

Supplementary Fig. 19 Functional analysis of novel/unannotated genes. a, **Gene Ontology** enrichment analysis of genes within WGCNA co-expression Module1 and Module2. BP, biological process; CC, cellular component and MF, molecular function. **b**,

Phylogenetic analysis of *zeat1* (left) and *zeat2* (right). *zeat1* and its potential homolog gene *bmb* are placed in two clades with corresponding orthologous genes from other fish species. See Supplementary Table 4 for the detailed sequence information used in the analysis. Phylogeny and domain structure of *zeat2* and its homologous genes from human and mouse. AA, amino acid. Local support values (1,000 times of resampling) were used for tree construction. **c**, Diagram showing how to obtain zygotic and maternal-zygotic mutants in this study. **d**, CRISPR-Cas9 knockout of *zeat1* (left) and *zeat2* (right). Diagram to show the gRNA design, mutation patterns and predict protein products with complete or truncated domains between the mutants and wild type. Sanger sequencing results of PCR products to validate the mutants obtained by genome editing. **e**, WT embryos were co-injected with *zeat1*-GFP mRNA and WGA-Alexa 647. The Zeat1-GFP fusion protein was observed to localize at the nuclear envelope, as indicated by the colocalization of the Zeat1-GFP signal with WGA-Alexa 647. **f**, The structure and gene annotation of zebrafish mitochondrial genome. The arrows indicate the location of primers for qRT-PCR.

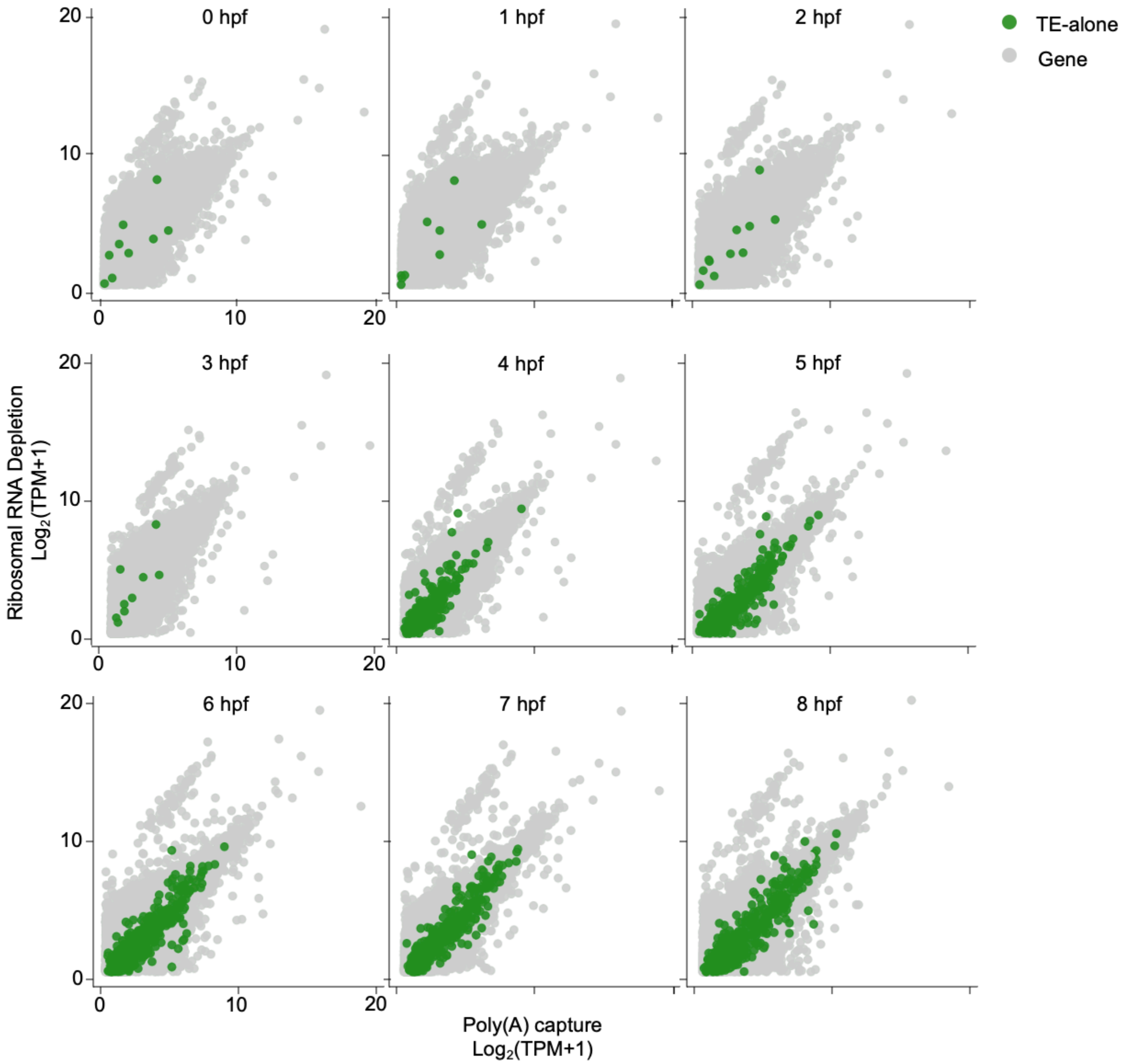

Supplementary Fig. 20 Quantification difference between two library preparations (Ribosomal RNA depletion and poly(A) enrichment) for mRNA sequencing. Each dot in the plot represents a transcript identified in this study. x-lab represents the $\log_2$ transformed TPM quantified by poly(A) capture library preparation, and y-lab represents the $\log_2$ transformed TPM quantified by ribosomal RNA depletion method. Compared to regular genes, TE-alone transcript abundance estimation has a good correlation between these two methods.

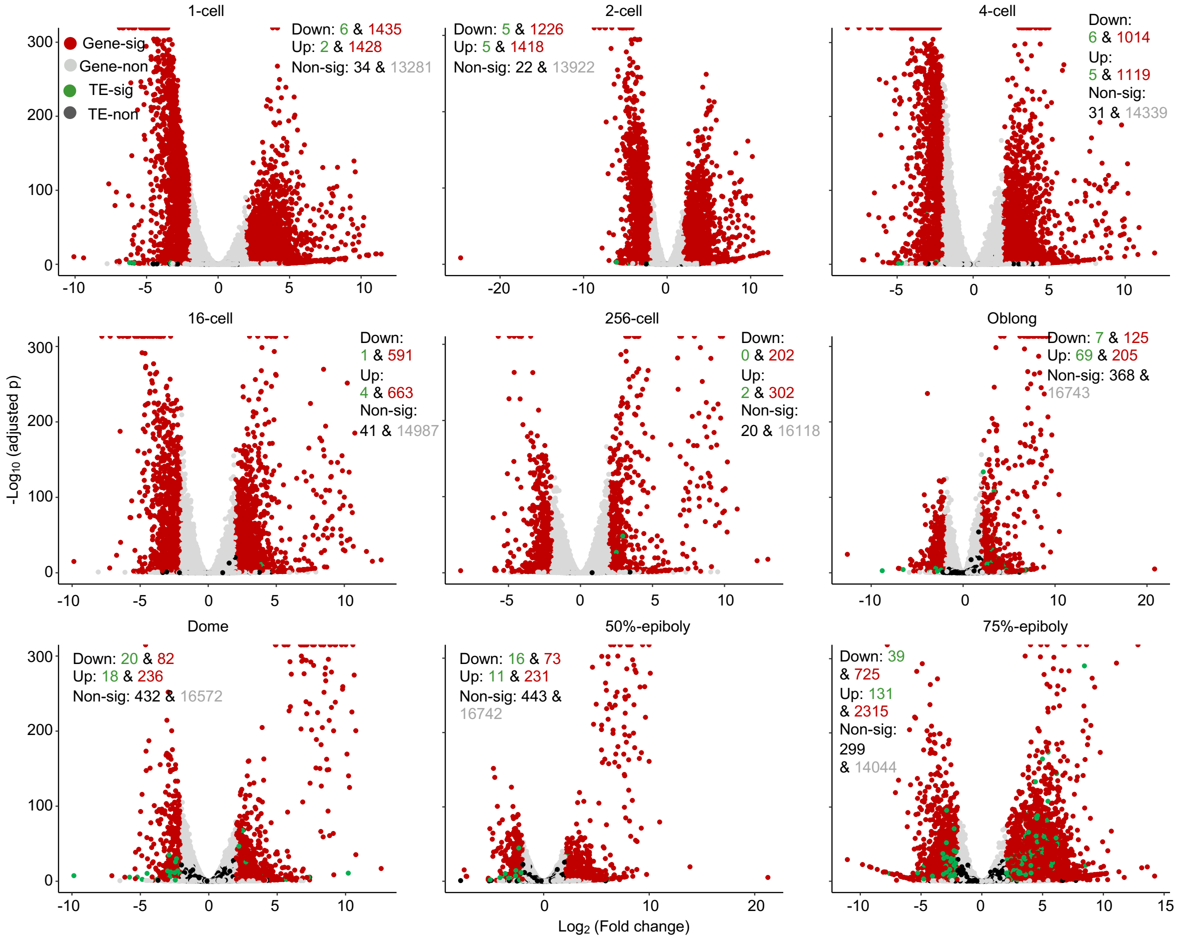

Supplementary Fig. 21. Volcano plots at each developmental stage. Differential analysis between poly(A) enrichment and rRNA depletion library preparation methods. Gene-sig: significantly differential expressed genes are highlighted in red color; Gene-non: insignificantly differential expressed genes are highlighted in grey color; TE-sig: significantly differential expressed TE-alone are highlighted in green color; and TE-non: insignificantly differential expressed genes are highlighted in black color. Down: down-regulated TE and genes in poly(A) enrichment vs. rRNA depletion, which are potentially caused by cytoplasmic polyadenylation; Up: down-regulated TE and genes in poly(A) enrichment vs. rRNA depletion and Non-sig: TE and genes with no significant changes. The counts are listed for each stage (TE & gene).

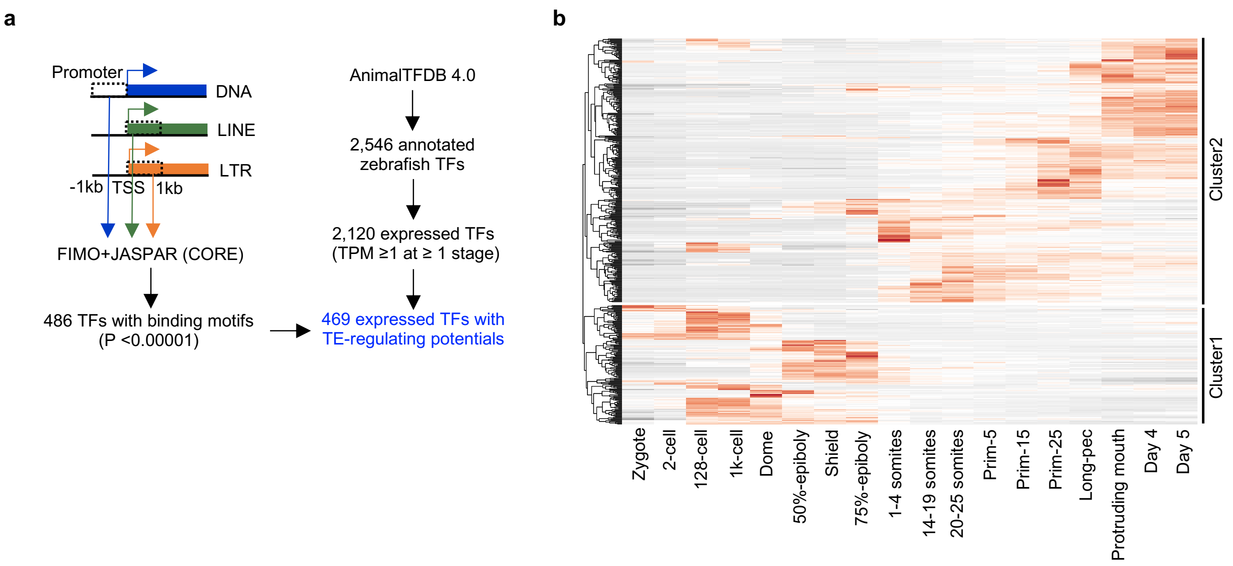

Supplementary Fig. 22 The prediction of TFs in TE regulation during early development in zebrafish. a, Pipeline for TF prediction; **b**, The expression pattern of 469 TFs during early embryonic development. Cluster1 represents TFs that are highly expressed until 75%-epiboly as TEs are highly activated, and cluster2 contains TFs that are highly expressed later during the repression period of ZTA.

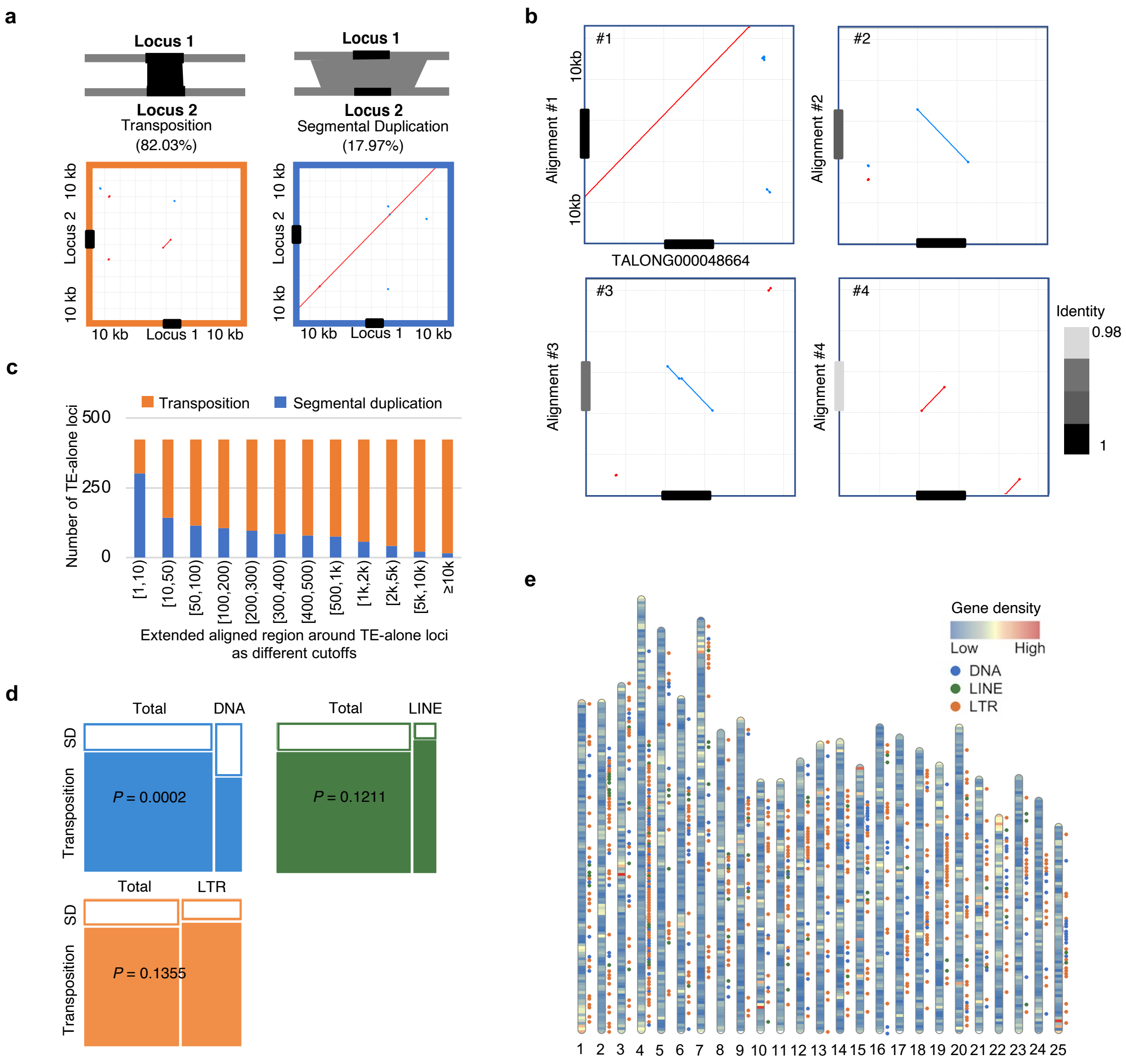

Supplementary Fig. 23 Active TE-alone loci are mainly originated by transposition. **a**, Chart to illustrate how to detect transposition (where only the TEs show high similarity) or segmental duplication (where both the TE regions and the surrounding regions show high similarity). Dot plots show typical patterns for these two mechanisms. The proportions for the two mechanisms are calculated using a 500 bp cutoff for surrounding regions. **b**, Example of choosing the most reliable donor TE-alone locus. An expressed TE-alone locus, TALONG000048664, has four homologous regions (alignments) in zebrafish genome. Based on the alignment quality (sequence identity), we consider the region with the best alignment quality as the most possible TE donor (alignment #1). Therefore, the possible origin mechanism can be inferred from the alignment #1. **c**, Different cutoffs for extended aligned regions to show that transposition is always the dominant mechanism for TE amplification. **d**, Chi-square test to show the different impact on DNA transposon, LTR and LINE by segmental duplication (SD) or transposition. **e**, Genomic distribution of active TE-alone loci along 25 zebrafish chromosomes. Each colored dot represents one active TE-alone locus. Two SINE loci are excluded.

Supplementary Tables 1-4

Supplementary Table 1. Summary of chimeric TE-alone transcripts

TE-alone transcript	Major TE subfamily	Proportion of major TE	Number of Minor TEs	Formation type
TALONT000987572	L2-3_DRe	0.967	1	Readthrough
TALONT001130078	L2-3_DRe	0.974	1	Readthrough
ENSDART00000139978	DNA2-8_DR	0.857	1	Readthrough
TALONT001528230	Gypsy172	0.982	1	Readthrough
TALONT001950926	Gypsy172	0.982	1	Readthrough
ENSDART00000134496	Gypsy-24	0.284	8	Readthrough
TALONT000193757	Harbinger-6_DR	0.663	2	Readthrough
TALONT000677847	ERV2	0.891	1	Readthrough
TALONT001247436	L2-1_DR	0.978	1	Readthrough
ENSDART00000159363	Nimb-2_DR	0.978	1	Readthrough
ENSDART00000158200	Tx1-5_DR	0.965	1	Readthrough
TALONT001275871	hAT-N114_DR	0.473	2	Readthrough
TALONT001220836	ERV1-N3	0.934	1	Readthrough
TALONT001563042	ERV1-N3	0.892	1	Readthrough
TALONT001532320	ERV1-N3B	0.686	1	Readthrough
TALONT001476450	DNA2-5_DR	0.878	1	Readthrough
TALONT001439999	BHIKHARI-5	0.791	2	Readthrough
TALONT000248167	EnSpm-14N1_DR	0.812	1	Readthrough
TALONT000492244	ERV1-N3B	0.686	1	Readthrough
TALONT000605596	DNA-8-13_DR	0.913	1	Readthrough
TALONT000613855	ERV1-N3B	0.686	1	Readthrough
TALONT000964064	ERV2	0.584	2	Readthrough
TALONT001196856	BHIKHARI	0.697	3	Readthrough
TALONT000922449	ERV1-N3B	0.688	1	Readthrough
TALONT001039427	ERV1-N3	0.985	1	Readthrough
TALONT001443784	ERV1-N3	0.739	1	Readthrough
TALONT001250195	DNA9TA1_DR	0.763	3	Readthrough
TALONT000953935	Gypsy-211	0.473	2	Readthrough
TALONT000957950	Gypsy152B	0.899	1	Readthrough
TALONT000964076	Gypsy-171	0.899	2	Readthrough
TALONT001688272	ERV1-10	0.977	1	Readthrough
TALONT001020257	Gypsy-171	0.793	1	Readthrough
TALONT001069620	ERV1-N3B	0.623	1	Readthrough
TALONT001123023	BHIKHARI-3	0.476	2	Readthrough
TALONT001678008	Gypsy161	0.912	1	Readthrough
TALONT001247261	ERV1-N3B	0.778	1	Readthrough
TALONT001281390	Gypsy65	0.99	1	Readthrough
TALONT001342088	Gypsy152B	0.898	1	Readthrough
TALONT001343365	Gypsy-24	0.779	1	Readthrough
TALONT001044702	BEL-26	0.927	1	TE insertion
TALONT001044703	BEL-26	0.927	1	TE insertion
TALONT000837694	ERV2	0.958	1	TE insertion
TALONT001092959	DNA-1-1_DR	0.765	1	TE insertion
TALONT000569167	ERV1-4	0.802	1	TE insertion
TALONT001299924	Gypsy152B	0.898	1	TE insertion
TALONT001156877	EnSpm-N3B_DR	0.883	1	TE insertion
TALONT000901747	Gypsy-171	0.815	1	TE insertion
TALONT001356955	Gypsy-219	0.843	1	TE insertion

Supplementary Table 2. Summary of transcript isoforms and exon numbers among different types of TEs

TE type	Class	Count
Transcript isoform number[1]		
DNA transposon	Multiple	40
DNA transposon	Single	87
LTR	Multiple	38
LTR	Single	304
LINE	Multiple	7
LINE	Single	72
Exon number[2]		
DNA transposon	Multiple	11
DNA transposon	Single	215
LTR	Multiple	224
LTR	Single	166
LINE	Multiple	73
LINE	Single	15

Note: 1) The count of isoform number is based on 548 TE-only loci (two SINE loci are excluded).
2) The exon number is calculated based on 704 TE-only transcripts (two SINE transcripts are excluded).

Supplementary Table 3. Core domain loss in different types of transposable elements

TE type	Total transcript	Total transcripts with ORFs	Total transcripts with domain loss with ORFs/without ORFs	Proportion of transcripts with domain loss (%)
DNA transposon	226	131	83/95	78.76
LINE	88	88	13/0	14.77
LTR	390	315	291/75	93.85
ERV	170	101	101/69	100.00

Supplementary Table 4. Details for homologous genes of *zeat1* for phylogeny construction.

NCBI reference sequence ID	NCBI Definition	Species
NP_001257483.1	protein brambleberry precursor	Danio rerio
XP_048013472.1	protein brambleberry isoform X4	Megalobrama amblycephala
XP_048013470.1	protein brambleberry isoform X3	Megalobrama amblycephala
KAF4095413.1	hypothetical protein G5714_024491	Onychostoma macrolepis
XP_016350013.1	PREDICTED: protein brambleberry	Sinocyclocheilus anshuiensis
KTG46222.1	hypothetical protein cypCar_00014491	Cyprinus carpio
XP_018923856.2	protein brambleberry-like	Cyprinus carpio
XP_048013469.1	protein brambleberry isoform X2	Megalobrama amblycephala
XP_016378109.1	PREDICTED: protein brambleberry isoform X2	Sinocyclocheilus rhinocerous
XP_042609535.1	protein brambleberry-like isoform X1	Cyprinus carpio
XP_026124937.1	uncharacterized protein LOC113107028	Carassius auratus
KAI2662070.1	Protein brambleberry	Labeo rohita
XP_039521821.1	uncharacterized protein LOC120475127 isoform X2	Pimephales promelas
XP_043102264.1	uncharacterized protein LOC122350109	Puntigrus tetrazona
XP_039521816.1	uncharacterized protein LOC120475127 isoform X1	Pimephales promelas
XP_016089552.1	PREDICTED: uncharacterized protein LOC107552217	Sinocyclocheilus grahami
XP_016323787.1	PREDICTED: uncharacterized protein LOC107674346	Sinocyclocheilus anshuiensis
XP_016411723.1	PREDICTED: uncharacterized protein LOC107743114	Sinocyclocheilus rhinocerous
KTG46871.1	hypothetical protein cypCar_00023052	Cyprinus carpio

Reference

1. Kapitonov, V.V. & Jurka, J. A novel class of SINE elements derived from 5S rRNA. *Mol Biol Evol* **20**, 694-702 (2003).
2. Lanciano, S. & Cristofari, G. Measuring and interpreting transposable element expression. *Nat Rev Genet* **21**, 721-736 (2020).
3. Chang, N.C., Rovira, Q., Wells, J., Feschotte, C. & Vaquerizas, J.M. Zebrafish transposable elements show extensive diversification in age, genomic distribution, and developmental expression. *Genome Res* **32**, 1408-1423 (2022).
4. Sakashita, A. *et al.* Endogenous retroviruses drive species-specific germline transcriptomes in mammals. *Nat Struct Mol Biol* **27**, 967-977 (2020).
5. Roy, S.W. The origin of recent introns: transposons? *Genome Biol* **5**, 251 (2004).
6. Roy, S.W. & Gilbert, W. The evolution of spliceosomal introns: patterns, puzzles and progress. *Nat Rev Genet* **7**, 211-21 (2006).
7. Schulz, K.N. & Harrison, M.M. Mechanisms regulating zygotic genome activation. *Nat Rev Genet* **20**, 221-234 (2019).
8. He, J. *et al.* Identifying transposable element expression dynamics and heterogeneity during development at the single-cell level with a processing pipeline scTE. *Nat Commun* **12**, 1456 (2021).
9. Svoboda, P. *et al.* RNAi and expression of retrotransposons MuERV-L and IAP in preimplantation mouse embryos. *Dev Biol* **269**, 276-85 (2004).
10. Wilson, C.A. *et al.* Wild sex in zebrafish: loss of the natural sex determinant in domesticated strains. *Genetics* **198**, 1291-308 (2014).
11. Suurväli, J. *et al.* The Laboratory Domestication of Zebrafish: From Diverse Populations to Inbred Substrains. *Mol Biol Evol* **37**, 1056-1069 (2020).
12. Pálfy, M., Schulze, G., Valen, E. & Vastenhouw, N.L. Chromatin accessibility established by Pou5f3, Sox19b and Nanog primes genes for activity during zebrafish genome activation. *PLoS Genet* **16**, e1008546 (2020).
13. Almeida, M.V., Vernaz, G., Putman, A.L.K. & Miska, E.A. Taming transposable elements in vertebrates: from epigenetic silencing to domestication. *Trends Genet* **38**, 529-553 (2022).
14. Giraldez, A.J. *et al.* Zebrafish MiR-430 promotes deadenylation and clearance of maternal mRNAs. *Science* **312**, 75-9 (2006).
15. Yao, Y. *et al.* Systematic characterization of small RNAome during zebrafish early developmental stages. *BMC Genomics* **15**, 117 (2014).
16. Houwing, S. *et al.* A role for Piwi and piRNAs in germ cell maintenance and transposon silencing in Zebrafish. *Cell* **129**, 69-82 (2007).
17. Stein, C.B. *et al.* Decoding the 5' nucleotide bias of PIWI-interacting RNAs. *Nat Commun* **10**, 828 (2019).
18. Wang, W. *et al.* The initial uridine of primary piRNAs does not create the tenth adenine that Is the hallmark of secondary piRNAs. *Mol Cell* **56**, 708-16 (2014).
19. Aanes, H. *et al.* Zebrafish mRNA sequencing deciphers novelties in transcriptome dynamics during maternal to zygotic transition. *Genome Res* **21**, 1328-38 (2011).

20. Winata, C.L. *et al.* Cytoplasmic polyadenylation-mediated translational control of maternal mRNAs directs maternal-to-zygotic transition. *Development* **145**(2018).
21. Hendrickson, P.G. *et al.* Conserved roles of mouse DUX and human DUX4 in activating cleavage-stage genes and MERVL/HERVL retrotransposons. *Nat Genet* **49**, 925-934 (2017).
22. Kinisu, M. *et al.* Klf5 establishes bi-potential cell fate by dual regulation of ICM and TE specification genes. *Cell Rep* **37**, 109982 (2021).
23. Yang, P., Wang, Y. & Macfarlan, T.S. The Role of KRAB-ZFPs in Transposable Element Repression and Mammalian Evolution. *Trends Genet* **33**, 871-881 (2017).
24. Imbeault, M., Helleboid, P.Y. & Trono, D. KRAB zinc-finger proteins contribute to the evolution of gene regulatory networks. *Nature* **543**, 550-554 (2017).
25. Wells, J.N. *et al.* Transposable elements drive the evolution of metazoan zinc finger genes. *Genome Res* **33**, 1325-1339 (2023).
26. Brouha, B. *et al.* Hot L1s account for the bulk of retrotransposition in the human population. *Proc Natl Acad Sci U S A* **100**, 5280-5 (2003).
27. Kano, H. *et al.* L1 retrotransposition occurs mainly in embryogenesis and creates somatic mosaicism. *Genes Dev* **23**, 1303-12 (2009).
28. Anderson, J.L. *et al.* Multiple sex-associated regions and a putative sex chromosome in zebrafish revealed by RAD mapping and population genomics. *PLoS One* **7**, e40701 (2012).
29. Xu, C. *et al.* Nanog-like regulates endoderm formation through the Mxtx2-Nodal pathway. *Dev Cell* **22**, 625-38 (2012).
