# Supplementary material for: Zygotic activation of transposable elements during zebrafish early embryogenesis
Source: Nat Commun. 2025 Apr 18;16:3692. doi: 10.1038/s41467-025-58863-7 (PMC12006353; doi:10.1038/s41467-025-58863-7)
Supplement: Supplementary file 2 — Description of Additional Supplementary Files [file 41467_2025_58863_MOESM2_ESM.pdf]

Description of Additional Supplementary Files

Supplementary Datasets

Supplementary Data1 Annotation results of TE-alone transcripts

Supplementary Data 2 Annotation results of TE-gene transcripts

Supplementary Data 3 Annotation results of gene transcripts

Supplementary Data 4 Quantification results of TE-alone transcripts

Supplementary Data 5 Quantification results of TE-gene transcripts

Supplementary Data 6 Quantification results of gene transcripts

Supplementary Data7 Pfam domain prediction on TE-alone transcripts

Supplementary Data 8 Summary of the number and total abundance of expressed transcripts at

each developmental stage

Supplementary Data 9 RNA-seq quantification of genes and TEs between wild type and mutants or

treatments

Supplementary Data 10 Transcript abundance in nuclear and cytosolic locations

Supplementary Data 11 Transcript abundance of transcript determined by short-read RNA-seq data

Supplementary Data 12 LTR annotation

Supplementary Data 13 Public data used in this study

Supplementary Data 14 Public software used in this study

Supplementary Data 15 In situ hybridization probes used in this study

Supplementary Data 16 TPM of TE-regulating TFs during early developmentin zerafish

Supplementary Movies

Supplementary Movie1: The Movie showing the continuous mitosis process in wild type zebrafish embryos during the cell cycle from the 64-cell to the 512-cell stages.

Supplementary Movie2: The Movie showing the mitotic process in Mzeat1 mutant zebrafish embryos during the cell cycle from the 64-cell to the 512-cell stages. Disrupted nuclear envelope fusion was observed, which leads to the formation of multinucleated cells during the transition.
