# Supplementary material for: Zygotic activation of transposable elements during zebrafish early embryogenesis
Source: Nat Commun. 2025 Apr 18;16:3692. doi: 10.1038/s41467-025-58863-7 (PMC12006353; doi:10.1038/s41467-025-58863-7)
Supplement: Supplementary file 6 — Reporting Summary [file 41467_2025_58863_MOESM6_ESM.pdf]

nature portfolio

Corresponding author(s): Yun-Gui Yang, Shunji Jia and Kin Fai Au

Last updated by author(s): Mar 2, 2025

Reporting Summary

Nature Portfolio wishes to improve the reproducibility of the work that we publish. This form provides structure for consistency and transparency in reporting. For further information on Nature Portfolio policies, see our Editorial Policies and the Editorial Policy Checklist.

Statistics

For all statistical analyses, confirm that the following items are present in the figure legend, table legend, main text, or Methods section.

n/a	Confirmed	
☐	☒	The exact sample size (*n*) for each experimental group/condition, given as a discrete number and unit of measurement
☐	☒	A statement on whether measurements were taken from distinct samples or whether the same sample was measured repeatedly
☐	☒	The statistical test(s) used AND whether they are one- or two-sided<br>*Only common tests should be described solely by name; describe more complex techniques in the Methods section.*
☒	☐	A description of all covariates tested
☒	☐	A description of any assumptions or corrections, such as tests of normality and adjustment for multiple comparisons
☐	☒	A full description of the statistical parameters including central tendency (e.g. means) or other basic estimates (e.g. regression coefficient) AND variation (e.g. standard deviation) or associated estimates of uncertainty (e.g. confidence intervals)
☐	☒	For null hypothesis testing, the test statistic (e.g. *F*, *t*, *r*) with confidence intervals, effect sizes, degrees of freedom and *P* value noted<br>*Give P values as exact values whenever suitable.*
☒	☐	For Bayesian analysis, information on the choice of priors and Markov chain Monte Carlo settings
☒	☐	For hierarchical and complex designs, identification of the appropriate level for tests and full reporting of outcomes
☐	☒	Estimates of effect sizes (e.g. Cohen's *d*, Pearson's *r*), indicating how they were calculated

Our web collection on statistics for biologists contains articles on many of the points above.

Software and code

Policy information about availability of computer code

Data collection

Sequencing was performed using both PacBio sequel and sequel II and Illumina HiSeq X Ten system with a 2×75 bp paired-end mode.
Zebrafish reference genome (GRCz11) and gene annotation (v103) were obtained from Ensembl.
Mouse reference genome (GRCm39) and gene annotation (107) were obtained from Ensembl.
Other public available data used in this study were downloaded from NCBI SRA.

Data analysis

Custom code developed in this study at https://github.com/Augroup/aTEA.
Public software is listed:
1. BamTools (v2.5.1) (https://github.com/pezmaster31/bamtools): convert Iso-seq data from BAM to Fasta
2. Iso-seq3 (https://github.com/pacificbiosciences/isoseq/): process raw PacBio subreads and CCS reads
3. minimap2 (v2.24): align long reads (CCS reads or transcripts) onto reference genome
4. TALON (v5.0): transcript identification
5. CD-HIT (v4.8.1): merge highly identical isoforms
6. RepeatMasker (v4.1.0) (https://www.repeatmasker.org): TE annotation
7. Integrated Genome Viewer (IGV) (v2.16.0): visualize the transcript identification results and manual curation
8. HISAT2 (v2.1.0): short-read RNA-seq data alignment
9. SAMtools (v1.9): convert SAM format into BAM format
10. StringTie (v1.3.5): transcript identification and quantification
11. BEDTools (v2.30.0): identify overlap between two bed files
12. TransDecoder (https://github.com/TransDecoder/TransDecoder): predict open reading frame from transcript sequences
13. HMMER (v3.3.1): predict conserved domain with protein sequences

14. MAFFT (v7.455): multiple sequences alignment
15. RSEM (v1.3.3): short-read based quantification
16. CIS-BP (http://cisbp.ccbr.utoronto.ca/): transcript factor binding site prediction
17. MEME suite: transcript factor binding site prediction
18. Cutadapt (v1.18): remove adapters for NGS data
19. Mview: visualize the multiple alignment results
20. BWA: align whole genome sequencing data onto zebrafish reference
21. deepTools (v2.0): visualize the results for epigenetic data
22. Bowtie (v1.3.1): align ChIP-seq data and small RNA data
23. Bowtie2 (v2.5.1): analyze ATAC-seq data
24. WebLogo 3: visualize the sequence motif
25. LTR_retriever: de novo identification of LTRs
26. BLASTN (v2.13.0): genome-wide homolog searching
27. MUMMER (v3.23): multiple alignment and generate dot plot for visualization
28. WGCNA (v1.72.1): coexpression network analysis
29. kallisto (v0.46.0): short reads based quantification on gene expression
30. DESeq2 (v1.38.3): identify differentially expressed genes
31. Cytoscape (v3.5.1): gene network analysis
32. DAVID (https://david.ncifcrf.gov/tools.jsp): gene functional enrichment analysis
33. FastTree (v2.1.11): phylogenetic analysis
34. iTOL (https://itol.embl.de/upload.cgi): draw phylogenetic tree
35. CHOPCHOP (v3): guide RNA designing for CRISPR-Cas9-mediated KO
36. R (v4.2.3): main tool for data visualization
37. Salmon (1.10.0): short-read RNA-seq quantification on transcript level
38. InterProScan (v5.61-93.0): domain and functional annotation
39. CPC: predicting the protein coding potential

For manuscripts utilizing custom algorithms or software that are central to the research but not yet described in published literature, software must be made available to editors and reviewers. We strongly encourage code deposition in a community repository (e.g. GitHub). See the Nature Portfolio guidelines for submitting code & software for further information.

Data

Policy information about availability of data

All manuscripts must include a data availability statement. This statement should provide the following information, where applicable:

- Accession codes, unique identifiers, or web links for publicly available datasets
- A description of any restrictions on data availability
- For clinical datasets or third party data, please ensure that the statement adheres to our policy

Data availability
All original sequencing data generated in this study have been submitted to the NCBI Sequence Read Archive (SRA) and are accessible under the BioProject accession number PRJNA1028258. The previously published data with the detailed description used in this study is listed in Supplementary Data. A video presentation introducing this project is available on the website (https://youtu.be/Bb-qBZZNQo4). Source data are provided with this paper (Supplementary Data).
Public data:
Species Data Type Tissue/Stage Accession # Platform References Hyperlink
Mouse Long read RNA-seq data Y. Qiao et al. https://www.ncbi.nlm.nih.gov/bioproject/PRJNA577068/
sperm cells SRR10267008 PACBIO_SMRT
oocyte cells SRR10267009 PACBIO_SMRT
1cell cells SRR10267010 PACBIO_SMRT
2cell cells SRR10267011 PACBIO_SMRT
4cell cells SRR10267012 PACBIO_SMRT
8cell cells SRR10267013 PACBIO_SMRT
blastocyst cells SRR10267014 PACBIO_SMRT
Short read RNA-seq data Y. Qiao et al. https://www.ncbi.nlm.nih.gov/bioproject/PRJNA577068/
sperm cells SRR10266988-SRR10266991 Illumina NovaSeq 6000
oocyte cells SRR10266992-SRR10266994 Illumina NovaSeq 6000
1cell cells SRR10266995-SRR10266997 Illumina NovaSeq 6000
2cell cells SRR10266998-SRR10267000 Illumina NovaSeq 6000
4cell cells SRR10267001,SRR10267002 Illumina NovaSeq 6000
8cell cells SRR10267003-SRR10267005 Illumina NovaSeq 6000
blastocyst cells SRR10267006 Illumina NovaSeq 6000

Single-embryo RNA-seq data M. Asami et al. https://www.ncbi.nlm.nih.gov/bioproject/PRJNA662943/
0h_rep1 SRR12624495 Illumina HiSeq 4000
0h_rep2 SRR12624508 Illumina HiSeq 4000
0h_rep3 SRR12624520 Illumina HiSeq 4000
0h_rep4 SRR12624526 Illumina HiSeq 4000
0h_rep5 SRR12624533 Illumina HiSeq 4000
0h_rep6 SRR12624540 Illumina HiSeq 4000
0h_rep6 SRR12624541 Illumina HiSeq 4000
2h_rep1 SRR12624496 Illumina HiSeq 4000
2h_rep2 SRR12624503 Illumina HiSeq 4000
2h_rep3 SRR12624509 Illumina HiSeq 4000
2h_rep4 SRR12624514 Illumina HiSeq 4000

2h_rep4 SRR12624515 Illumina HiSeq 4000
2h_rep5 SRR12624521 Illumina HiSeq 4000
2h_rep6 SRR12624527 Illumina HiSeq 4000
2h_rep7 SRR12624534 Illumina HiSeq 4000
2h_rep8 SRR12624542 Illumina HiSeq 4000
2h_rep8 SRR12624543 Illumina HiSeq 4000
4h_rep1 SRR12624497 Illumina HiSeq 4000
4h_rep2 SRR12624504 Illumina HiSeq 4000
4h_rep3 SRR12624510 Illumina HiSeq 4000
4h_rep4 SRR12624516 Illumina HiSeq 4000
4h_rep5 SRR12624522 Illumina HiSeq 4000
4h_rep6 SRR12624528 Illumina HiSeq 4000
4h_rep7 SRR12624535 Illumina HiSeq 4000
4h_rep8 SRR12624544 Illumina HiSeq 4000
4h_rep8 SRR12624545 Illumina HiSeq 4000
6h_rep1 SRR12624498 Illumina HiSeq 4000
6h_rep2 SRR12624505 Illumina HiSeq 4000
6h_rep3 SRR12624511 Illumina HiSeq 4000
6h_rep4 SRR12624517 Illumina HiSeq 4000
6h_rep5 SRR12624523 Illumina HiSeq 4000
6h_rep6 SRR12624529 Illumina HiSeq 4000
6h_rep7 SRR12624536 Illumina HiSeq 4000
6h_rep8 SRR12624546 Illumina HiSeq 4000
6h_rep8 SRR12624547 Illumina HiSeq 4000
8h_rep1 SRR12624499 Illumina HiSeq 4000
8h_rep2 SRR12624512 Illumina HiSeq 4000
8h_rep3 SRR12624518 Illumina HiSeq 4000
8h_rep4 SRR12624530 Illumina HiSeq 4000
8h_rep5 SRR12624537 Illumina HiSeq 4000
8h_rep6 SRR12624548 Illumina HiSeq 4000
8h_rep6 SRR12624549 Illumina HiSeq 4000
10h_rep1 SRR12624500 Illumina HiSeq 4000
10h_rep2 SRR12624502 Illumina HiSeq 4000
10h_rep3 SRR12624506 Illumina HiSeq 4000
10h_rep4 SRR12624524 Illumina HiSeq 4000
10h_rep5 SRR12624531 Illumina HiSeq 4000
10h_rep6 SRR12624538 Illumina HiSeq 4000
10h_rep7 SRR12624550 Illumina HiSeq 4000
10h_rep7 SRR12624551 Illumina HiSeq 4000
12h_rep1 SRR12624501 Illumina HiSeq 4000
12h_rep2 SRR12624507 Illumina HiSeq 4000
12h_rep3 SRR12624513 Illumina HiSeq 4000
12h_rep4 SRR12624519 Illumina HiSeq 4000
12h_rep5 SRR12624525 Illumina HiSeq 4000
12h_rep6 SRR12624532 Illumina HiSeq 4000
12h_rep7 SRR12624539 Illumina HiSeq 4000
12h_rep8 SRR12624552 Illumina HiSeq 4000
12h_rep8 SRR12624553 Illumina HiSeq 4000
Zebrafish RNA-seq data over zebrafish early development R. J. White et al. https://www.ncbi.nlm.nih.gov/bioproject/PRJNA529241/
Oblong SRR8788666 Illumina HiSeq 2000
Sphere SRR8788679 Illumina HiSeq 2000
Dome ERR1442621 Illumina HiSeq 2500
75%-epiboly ERR1442606 Illumina HiSeq 2500
Somite 1-4 ERR1442636 Illumina HiSeq 2500
Somite 14-19 ERR1442611 Illumina HiSeq 2500
Somite 20-25 ERR1442616 Illumina HiSeq 2500
Prim 5 ERR1442566 Illumina HiSeq 2500
Prim 15 ERR1442571 Illumina HiSeq 2500
Prim 25 ERR1442576 Illumina HiSeq 2500
Long-pec ERR1442581 Illumina HiSeq 2500
Protruding-mouth ERR1442586 Illumina HiSeq 2500
Day 4 ERR1442591 Illumina HiSeq 2500
Day 5 ERR1442596 Illumina HiSeq 2500
RNA-seq between wild type and treatments M. T. Lee et al. https://www.ncbi.nlm.nih.gov/bioproject/PRJNA206070/
WT_4hpf SRR870757-SRR870771 Illumina HiSeq 2000
WT_6hpf SRR870896-SRR870909 Illumina HiSeq 2000
WT_8hpf SRR959342-SRR959347 Illumina HiSeq 2000
α-Amanitin_4hpf SRR870772-SRR870783 Illumina HiSeq 2000
α-Amanitin_6hpf SRR870910-SRR870918 Illumina HiSeq 2000
CHX_4hpf SRR959304-SRR959319 Illumina HiSeq 2000
CHX_6hpf SRR870919-SRR870922 Illumina HiSeq 2000
triple_mutant_4hpf SRR870883-SRR870895 Illumina HiSeq 2000
triple_mutant_6hpf SRR959348-SRR959354 Illumina HiSeq 2000
triple_mutant_8hpf SRR959355-SRR959361 Illumina HiSeq 2000
triple_mutant_rescue_4hpf SRR959320-SRR959335 Illumina HiSeq 2000
triple_mutant_rescue_6hpf SRR959362-SRR959369 Illumina HiSeq 2000

triple_mutant_rescue_8hpf SRR959370-SRR959373 Illumina HiSeq 2000
RNA-seq from nuclei and cytoplasm over zebrafish early development S. Pillay, et al. https://www.ncbi.nlm.nih.gov/bioproject/PRJNA599208/
1k-cell_nuclear SRR10836780-SRR10836783 Illumina NextSeq 500
1k-cell_cytosolic SRR10836784-SRR10836787 Illumina NextSeq 500
Dome_nuclear SRR10836788-SRR10836791 Illumina NextSeq 500
Dome_cytosolic SRR10836792-SRR10836795 Illumina NextSeq 500
Shield_nuclear SRR10836796-SRR10836799 Illumina NextSeq 500
Shield_cytosolic SRR10836800-SRR10836803 Illumina NextSeq 500
Small RNA sequencing data Y. Yao et al. https://www.ncbi.nlm.nih.gov/bioproject/PRJNA215266/
1-cell SRR953087 Illumina HiSeq 2000
16-cell SRR953522 Illumina HiSeq 2000
512-cell SRR953530 Illumina HiSeq 2000
Oblong SRR953531 Illumina HiSeq 2000
50%-epiboly SRR953532 Illumina HiSeq 2000
6-somite SRR953567 Illumina HiSeq 2000
24 hpf SRR953568 Illumina HiSeq 2000
48 hpf SRR953569 Illumina HiSeq 2000
ATAC-seq data M. Palfy, et al https://www.ncbi.nlm.nih.gov/bioproject/PRJNA542075/
256-cell SRR9032650,SRR9032651 Illumina HiSeq 2500
High SRR9032662-SRR9032664 Illumina HiSeq 2500
Oblong SRR9032638-SRR9032640 Illumina HiSeq 2500
Sphere SRR9032674-SRR9032676 Illumina HiSeq 2500
Dome SRR9032658-SRR9032661 Illumina HiSeq 2500
Shield SRR9032668-SRR9032673 Illumina HiSeq 2500
80%-epiboly SRR9032652-SRR9032657 Illumina HiSeq 2500
ChIP-seq (H3K9me3) data K. Laue, et al https://www.ncbi.nlm.nih.gov/bioproject/PRJNA449956/
64-cell (2.5 hpf) Control SRR6996307, SRR6996309 Illumina NextSeq 500
64-cell (2.5 hpf) ChIP-seq SRR6996308,SRR6996310 Illumina NextSeq 500
Sphere (4.5 hpf) Control SRR6996303,SRR6996305 Illumina NextSeq 500
Sphere (4.5 hpf) ChIP-seq SRR6996304,SRR6996306 Illumina NextSeq 500
Shield (6 hpf) Control SRR6996299, SRR6996301 Illumina NextSeq 500
Shield (6 hpf) ChIP-seq SRR6996300,SRR6996302 Illumina NextSeq 500
ChIP-seq (H3K4me3) data1 Zhan, et al 2018 https://www.ncbi.nlm.nih.gov/bioproject/PRJNA473799/
Oocyte SRR7235500,SRR7235501,SRR7235502 HiSeq X Ten
Dome SRR7235519,SRR7235520,SRR7235521,SRR7235522,SRR7235523 HiSeq X Ten
ChIP-seq (H3K4me3) data2 Zhu, et al 2019 https://www.ncbi.nlm.nih.gov/bioproject/PRJNA434216/
Sperm SRR6729438,SRR6729439 HiSeq X Ten
Oocyte SRR6729440,SRR6729441 HiSeq X Ten
2-cell SRR6729442,SRR6729443 HiSeq X Ten
16-cell SRR6729444,SRR6729445 HiSeq X Ten
128-cell SRR6729446,SRR6729447 HiSeq X Ten
1k-cell SRR6729448,SRR6729449 HiSeq X Ten
ChIP-seq (H3K27ac) Zhan, et al 2018 https://www.ncbi.nlm.nih.gov/bioproject/PRJNA473799/
Oocyte SRR7235508,SRR7235509 HiSeq X Ten
256-cell SRR7235554,SRR7235555,SRR7235556 HiSeq X Ten
Dome SRR7235529,SRR7235530,SRR7235531 HiSeq X Ten

ChIP-seq (nanog+mxtx2) Xu, et al 2012 https://www.ncbi.nlm.nih.gov/bioproject/PRJNA156233/
3.5 hpf- nanog SRR392588 Illumina Genome Analyzer II
3.5 hpf- control SRR392591 Illumina Genome Analyzer II
4.5 hpf- nanog SRR392589 Illumina Genome Analyzer II
4.5 hpf- control SRR392592 Illumina Genome Analyzer II
4.5 hpf- mxtx2 SRR392590 Illumina Genome Analyzer II
4.5 hpf- control SRR392593 Illumina Genome Analyzer II
Genomic data
AB reference genome PRJEB38589 https://www.ncbi.nlm.nih.gov/bioproject/PRJEB38589/
Nadia reference genome PRJEB38577 https://www.ncbi.nlm.nih.gov/bioproject/PRJEB38577/
CB reference genome PRJEB38573 https://www.ncbi.nlm.nih.gov/bioproject/PRJEB38573/

Research involving human participants, their data, or biological material

Policy information about studies with human participants or human data. See also policy information about sex, gender (identity/presentation), and sexual orientation and race, ethnicity and racism.

Reporting on sex and gender

Use the terms sex (biological attribute) and gender (shaped by social and cultural circumstances) carefully in order to avoid confusing both terms. Indicate if findings apply to only one sex or gender; describe whether sex and gender were considered in study design; whether sex and/or gender was determined based on self-reporting or assigned and methods used. Provide in the source data disaggregated sex and gender data, where this information has been collected, and if consent has been obtained for sharing of individual-level data; provide overall numbers in this Reporting Summary. Please state if this information has not been collected. Report sex- and gender-based analyses where performed, justify reasons for lack of sex- and gender-based analysis.

Reporting on race, ethnicity, or

Please specify the socially constructed or socially relevant categorization variable(s) used in your manuscript and explain why they were used. Please note that such variables should not be used as proxies for other socially constructed/relevant variables

other socially relevant groupings	*(for example, race or ethnicity should not be used as a proxy for socioeconomic status). Provide clear definitions of the relevant terms used, how they were provided (by the participants/respondents, the researchers, or third parties), and the method(s) used to classify people into the different categories (e.g. self-report, census or administrative data, social media data, etc.) Please provide details about how you controlled for confounding variables in your analyses.*
Population characteristics	*Describe the covariate-relevant population characteristics of the human research participants (e.g. age, genotypic information, past and current diagnosis and treatment categories). If you filled out the behavioural & social sciences study design questions and have nothing to add here, write "See above."*
Recruitment	*Describe how participants were recruited. Outline any potential self-selection bias or other biases that may be present and how these are likely to impact results.*
Ethics oversight	*Identify the organization(s) that approved the study protocol.*

Note that full information on the approval of the study protocol must also be provided in the manuscript.

Field-specific reporting

Please select the one below that is the best fit for your research. If you are not sure, read the appropriate sections before making your selection.

☒ Life sciences ☐ Behavioural & social sciences ☐ Ecological, evolutionary & environmental sciences

For a reference copy of the document with all sections, see nature.com/documents/nr-reporting-summary-flat.pdf

Life sciences study design

All studies must disclose on these points even when the disclosure is negative.

Sample size	No sample size calculation was performed. For sequencing, >3 biological replicates are sufficient. For experiment, > 10 embryos were selected which should be sufficient.
Data exclusions	No data was excluded from the analyses.
Replication	Illumina short-read RNA-seq have eight replicates. Zebrafish embryos for phenotyping have the minimal number of 55. All attempts at replication were successful.
Randomization	The samples for data generation are randomly selected and pooled. In the time course analysis, samples at each time point were randomly selected for downstream analysis. For the comparison between mutant and wild type, corresponding samples were randomly selected for each group. We do not have any other complicate experiment designs which require sample randomization.
Blinding	The authors were not blinded to group allocation. Blind was not necessary for this study because the accurate determination of developmental stages was essential for the data generation and implementation. At the same developmental stage, embryos are randomly selected for data generation.

Reporting for specific materials, systems and methods

We require information from authors about some types of materials, experimental systems and methods used in many studies. Here, indicate whether each material, system or method listed is relevant to your study. If you are not sure if a list item applies to your research, read the appropriate section before selecting a response.

Materials & experimental systems

n/a	Involved in the study	
☐	☒	Antibodies
☒	☐	Eukaryotic cell lines
☒	☐	Palaeontology and archaeology
☐	☒	Animals and other organisms
☒	☐	Clinical data
☒	☐	Dual use research of concern
☒	☐	Plants

Methods

n/a	Involved in the study	
☒	☐	ChIP-seq
☒	☐	Flow cytometry
☒	☐	MRI-based neuroimaging

Antibodies

Antibodies used	1. Digoxigenin-AP antibody Catlog Nomber: 11093274910, supplier name: Roche<br>2. β-Catenin (L54E2) Mouse mAb, Catlog Nomber: 2677, supplier name: Cell Signaling Technology<br>3. Alexa Fluor 488 AffiniPure Goat anti-Mouse IgG , Catlog Nomber: 115-545-003, supplier name: Jackson ImmunoResearch Labs

Validation	β-Catenin (L54E2) Mouse mAb is validated using western blot, confocal immunofluorescent analysis and flow cytometric analysis.

Animals and other research organisms

Policy information about studies involving animals; ARRIVE guidelines recommended for reporting animal research, and Sex and Gender in Research

Laboratory animals	Two zebrafish strains, AB and India WT, were used in this study. Eleven developmental stages of embryos were defined at specific time points, and embryos were collected at the following stages: fertilized egg (immediately after fertilization), 1-cell at 0.5 hours post fertilization (hpf), 64-cell at 2, 1k-cell at 3, high at 3.3, oblong at 3.7, sphere at 4, dome at 4.3, 30%-epiboly at 4.7, 50%-epiboly at 5.3 and shield at 6 hpf, respectively. RNA isolated from 10-day after fertilization zebrafish using the miRNeasy RNA extraction kit.
Wild animals	The study did not involve with wild animals.
Reporting on sex	This information has not been collected.
Field-collected samples	The study did not involve with samples collected from the field.
Ethics oversight	All fish experiments in this study were approved by the animal care and use committee of the Institute of Genetics and Developmental Biology, Chinese Academy of Sciences.

Note that full information on the approval of the study protocol must also be provided in the manuscript.

Plants

Seed stocks	*Report on the source of all seed stocks or other plant material used. If applicable, state the seed stock centre and catalogue number. If plant specimens were collected from the field, describe the collection location, date and sampling procedures.*
Novel plant genotypes	*Describe the methods by which all novel plant genotypes were produced. This includes those generated by transgenic approaches, gene editing, chemical/radiation-based mutagenesis and hybridization. For transgenic lines, describe the transformation method, the number of independent lines analyzed and the generation upon which experiments were performed. For gene-edited lines, describe the editor used, the endogenous sequence targeted for editing, the targeting guide RNA sequence (if applicable) and how the editor was applied.*
Authentication	*Describe any authentication procedures for each seed stock used or novel genotype generated. Describe any experiments used to assess the effect of a mutation and, where applicable, how potential secondary effects (e.g. second site T-DNA insertions, mosiacism, off-target gene editing) were examined.*
